# Microbiome-driven breeding strategy potentially improves beef fatty acid profile benefiting human health and reduces methane emissions

**DOI:** 10.1186/s40168-022-01352-6

**Published:** 2022-10-05

**Authors:** Marina Martínez-Álvaro, Jennifer Mattock, Marc Auffret, Ziqing Weng, Carol-Anne Duthie, Richard J. Dewhurst, Matthew A. Cleveland, Mick Watson, Rainer Roehe

**Affiliations:** 1 Scotland’s Rural College, Edinburgh, UK; 2 The Roslin Institute and the Royal (Dick) School of Veterinary Studies, University of Edinburgh, Edinburgh, UK; 3 Agrifirm, Drongen, Belgium; 4 Genus plc, DeForest, WI USA

**Keywords:** Rumen microbiome, Beef, Conjugated linoleic acid, Very long-chain n-3 fatty acids, Microbial genes, Microbiome-driven breeding, Genomic selection, Methane emissions

## Abstract

**Background:**

Healthier ruminant products can be achieved by adequate manipulation of the rumen microbiota to increase the flux of beneficial fatty acids reaching host tissues. Genomic selection to modify the microbiome function provides a permanent and accumulative solution, which may have also favourable consequences in other traits of interest (e.g. methane emissions). Possibly due to a lack of data, this strategy has never been explored.

**Results:**

This study provides a comprehensive identification of ruminal microbial mechanisms under host genomic influence that directly or indirectly affect the content of unsaturated fatty acids in beef associated with human dietary health benefits C18:3n-3, C20:5n-3, C22:5n-3, C22:6n-3 or *cis-9*, *trans-11* C18:2 and *trans-11* C18:1 in relation to hypercholesterolemic saturated fatty acids C12:0, C14:0 and C16:0, referred to as N3 and CLA indices. We first identified that ~27.6% (1002/3633) of the functional core additive log-ratio transformed microbial gene abundances (*alr*-MG) in the rumen were at least moderately host-genomically influenced (HGFC). Of these, 372 *alr*-MG were host-genomically correlated with the N3 index (*n*=290), CLA index (*n*=66) or with both (*n*=16), indicating that the HGFC influence on beef fatty acid composition is much more complex than the direct regulation of microbial lipolysis and biohydrogenation of dietary lipids and that N3 index variation is more strongly subjected to variations in the HGFC than CLA. Of these 372 *alr*-MG, 110 were correlated with the N3 and/or CLA index in the same direction, suggesting the opportunity for enhancement of both indices simultaneously through a microbiome-driven breeding strategy. These microbial genes were involved in microbial protein synthesis (*aroF* and *serA*), carbohydrate metabolism and transport (*galT*, *msmX*), lipopolysaccharide biosynthesis (*kdsA*, *lpxD*, *lpxB*), or flagellar synthesis (*flgB*, *fliN*) in certain genera within the Proteobacteria phyla (e.g. *Serratia*, *Aeromonas*). A microbiome-driven breeding strategy based on these microbial mechanisms as sole information criteria resulted in a positive selection response for both indices (1.36±0.24 and 0.79±0.21 sd of N3 and CLA indices, at 2.06 selection intensity). When evaluating the impact of our microbiome-driven breeding strategy to increase N3 and CLA indices on the environmental trait methane emissions (g/kg of dry matter intake), we obtained a correlated mitigation response of −0.41±0.12 sd.

**Conclusion:**

This research provides insight on the possibility of using the ruminal functional microbiome as information for host genomic selection, which could simultaneously improve several microbiome-driven traits of interest, in this study exemplified with meat quality traits and methane emissions.

Video Abstract

**Supplementary Information:**

The online version contains supplementary material available at 10.1186/s40168-022-01352-6.

## Background

The human diet in industrialized countries is characterized by high amounts of hypercholesterolemic saturated fatty acids (C12:0, C14:0 and C16:0) [1–4], and low amounts of n-3 fatty acids [1], which prevent cardiovascular diseases [5] and have additional anti-inflammatory properties [6]. Beef contains more saturated fatty acids compared to pig or chicken meats [7, 8], explained by lipolysis and subsequent biohydrogenation of ingested UFA by the rumen microbiota. Assuming no marine supplements in the diet, the accumulation of long chain n-3 fatty acids in the muscle depends on the flux of C18:3n-3 biohydrogenation in the rumen and on the rate of C18:3n-3 endogenous elongation and desaturation [9] into very long chain n-3 (C20:5n-3, C22:5n-3, C22:6n-3) in body tissues through the action of very-long-chain fatty acid elongase and fatty acyl desaturase enzymes [10]. On the other hand, foods derived from ruminants account for more than 90% [11] of the total *cis*-*9*, *trans*-*11* C18:2 intake in the human diet, a very valuable molecule associated with major beneficial health effects (e.g. antitumor, antidiabetic, anti-inflammatory and anti-atherosclerotic properties) [7, 12, 13]. The content of *cis-9*, *trans-11* C18:2 in the body fats of ruminants derives from two sources: first, from endogenous synthesis via the enzymatic activity of stearyl-CoA desaturase [9, 14, 15], its concentration being related to the higher rumen efflux of *trans-11* C18:1 and second, from the direct absorption of *cis-9*, *trans-11* C18:2 from the duodenum, which is mainly produced in the first step of biohydrogenation of C18:2n-6 in the rumen, provided that further conversion to other C18:0 isomers is avoided [15, 16]. Therefore, healthier ruminant products can be achieved by adequate manipulation of the rumen microbiota aiming at reducing the final step of *cis-9*, *trans-11* C18:2 [17] biohydrogenation and increasing the bypass of C18:3n-3 [18], *trans-11* C18:1 and *cis-9*, *trans-11* C18:2 flux to the muscle.

Lipolysis activity (by lipases, phospholipases (A,C [19]), carboxylesterases and esterases [20, 21]) and biohydrogenation activity (e.g. by linoleate isomerases [15, 19] and octadienoate reductases) in the rumen is mainly performed by bacteria, as a defence against a toxic challenge [22]. As an example, *Anaerovibrio lipolytica* [23] hydrolyses triacylglycerol, and *Butyrivibrio*-like species hydrolyse phospholipids and galactolipids, and also play biohydrogenation activity (e.g. *Butyrivibrio fibrisolvens* [24–26] and *Butyrivibrio proteoclasticus* [19]), forming *cis-9*, *trans-11* C18:2 as an intermediate in the process [27]*.* The list of microorganisms with biohydrogenation or lipolytic activity in the rumen has now expanded to include species from the genera *Pseudomonas*, *Vibrio*, *Salmonella*, *Aeromonas*, *Serratia* [20, 21, 28], *Propionibacterium* [19], *Treponema* [29], *Bacillus* and *Clostridium* [30], among others*.* Lipolysis activity of protozoa also occurs in the rumen to a lesser extent [31, 32], but nevertheless significantly affects the flow of unsaturated fatty acids reaching the duodenum [33] and has been recently linked to the fatty acid profile of milk [34]. Similarly, anaerobic rumen fungi perform biohydrogenation, but the activity is negligible compared to that of *Butyrivibrio fibrisolvens* [35, 36]*.*

Lipolytic and biohydrogenative patterns in the rumen can be modified by inducing changes in the composition of the microbiota through dietary strategies (polyunsaturated fatty acid (P-UFA) intake or antimicrobial feed additives) [37–39]. In some cases, the success of these strategies may be compromised by the adaptation of the microbiota to the new environment [40] or negative effects on the sensory quality of the product [41]. In addition to dietary strategies, breeding-induced changes in the composition of the rumen microbiome could lead to permanent changes accumulated over generations of selection, as the animal’s genome influences part of the microbial colonization and function [42–53]. Modification of lipolysis and biohydrogenation of dietary lipids by this strategy has never been explored, but holds promise because the microbial functions and taxa associated are very sensitive [22, 54] to host characteristics influenced by host genomic factors (e.g. epithelial absorption of VFA affecting pH [46] or passaging rates [43]). In our previous study [53], we showed how genomic selection based on a subset of additive log-ratio transformed microbial gene abundances (*alr*-MG) can achieve a reduction in methane (CH_4_) emissions even greater than that achieved with the measured trait in respiration chambers, avoiding the high cost. A larger number of microbial functions showed a strong correlation to CH_4_ emissions in comparison to microbial taxa at the genus level and is therefore more informative as a selection criterion [53].

Targeting one microbial activity is likely to have consequences for others, although the secondary effects may not necessarily be detrimental. In nutritional studies, a substantial overlap has been observed between the inhibitory effects of long-chain poly-UFA on biohydrogenation and methanogenesis [39, 55, 56]. Also, microbial metabolic pathways that simultaneously affect fatty acid biohydrogenation and methanogenesis are likely to have a host genetic component, as shown by a divergent selection experiment for methane emissions in sheep, in which it was observed that the low-methane yield line had greater levels of fatty acids associated with the early stages of rumen biohydrogenation, such as *cis-9*, *trans-11* C18:2 and *trans-11* C18:1 [57]. Livestock contributes ~8–12% of anthropogenic emissions [58, 59], with one main contributor being enteric CH_4_ emissions from ruminants, which have a warming potential 28 times greater than CO_2_ [58], despite CH_4_ remaining in the atmosphere for much less time (12 versus >100 years) [60]. The beef industry faces the challenge of producing high-quality products while minimising environmental impact. Therefore, in this study, we extended our research by evaluating the impact of a microbiome-driven breeding strategy for improved meat quality on host CH_4_ emissions.

Through an extensive identification of the functional rumen microbiome of 359 animals, the first objective of this study is to elucidate which host-genomically influenced functional mechanisms in the rumen are associated with N3 and CLA indices in beef, defined as the proportion of beneficial fatty acids (long chain n-3 or *cis-9*, *trans-11* C18:2 and *trans-11* C18:1) in relation to hypercholesterolemic saturated fatty acids (C12:0, C14:0 and C16:0). To this objective, we are considering not only lipolytic/biohydrogenation activities but also other metabolic interdependencies (e.g. H_2_ metabolism). The second objective was to design a microbiome breeding strategy based on these microbial functions that, optimally, can simultaneously increase N3 and CLA indices in beef. The third objective was to evaluate the impact of this microbiome-driven breeding strategy on part of the environmental footprint of ruminants, measured as their CH_4_ emissions.

## Results

### Fatty acid indices are controlled by the host genome

Our research focuses on the development of a microbiome-driven breeding strategy to improve beef quality by increasing the content of long chain n-3 fatty acids (C18:3n-3, C20:5n-3, C22:5n-3 and C22:6n-3), *cis-9*, *trans-11* C18:2 and *trans*-11 C18:1 in beef in relation to the content of hypercholesterolemic saturated fatty acids (C12:0, C14:0 and C16:0). For this purpose, we propose the N3 index calculated as the natural log of (C18:3n-3 + C20:5n-3 + C22:5n-3 + C22:6n-3)/(C12:0 + C14:0 + C16:0) and the CLA index calculated as the natural log of (*cis-9*, *trans-11* C18:2 + *trans*-11 C18:1)/(C12:0 + C14:0 + C16:0) as breeding goal traits (Figure S1).

Substantial phenotypic variation between animals in N3 and CLA indices was due to variability in their estimated host genomic breeding values. Both traits exhibited high heritabilities (h^2^) (h^2^_N3_=0.76±0.16 and h^2^_CLA_=0.57±0.17) with strong statistical evidence for host genomic effects. This strong statistical evidence was ascertained by large Bayes Factors (BF) (1.81 × 10^+6^ for N3 and 5.55 × 10^+6^ for CLA) and large deviance information criterion differences between models with and without host genomic effects (DIC_diff_) (−320 for N3 and −129 for CLA). Despite the drawbacks associated with considering ratio traits as a breeding objective [61], fatty acid indices were used in this study because (1) they were adequately analysed considering the compositional nature of fatty acid data and (2) their biological implications and high h^2^ values provide key information about how the host genes affecting meat quality influence the rumen microbial metabolism of dietary lipids. In addition, our results suggest that a simultaneous increase of N3 and CLA indices in beef is favoured by their estimated positive host genomic correlation (rg_N3, CLA_=0.39, with probability of being different from 0 (P_0_) = 0.93).

### Microbial genes directly involved in lipolytic and biohydrogenation activity of dietary lipids in the rumen appear to be beyond the influence of the host genome

**Fig. 1:**
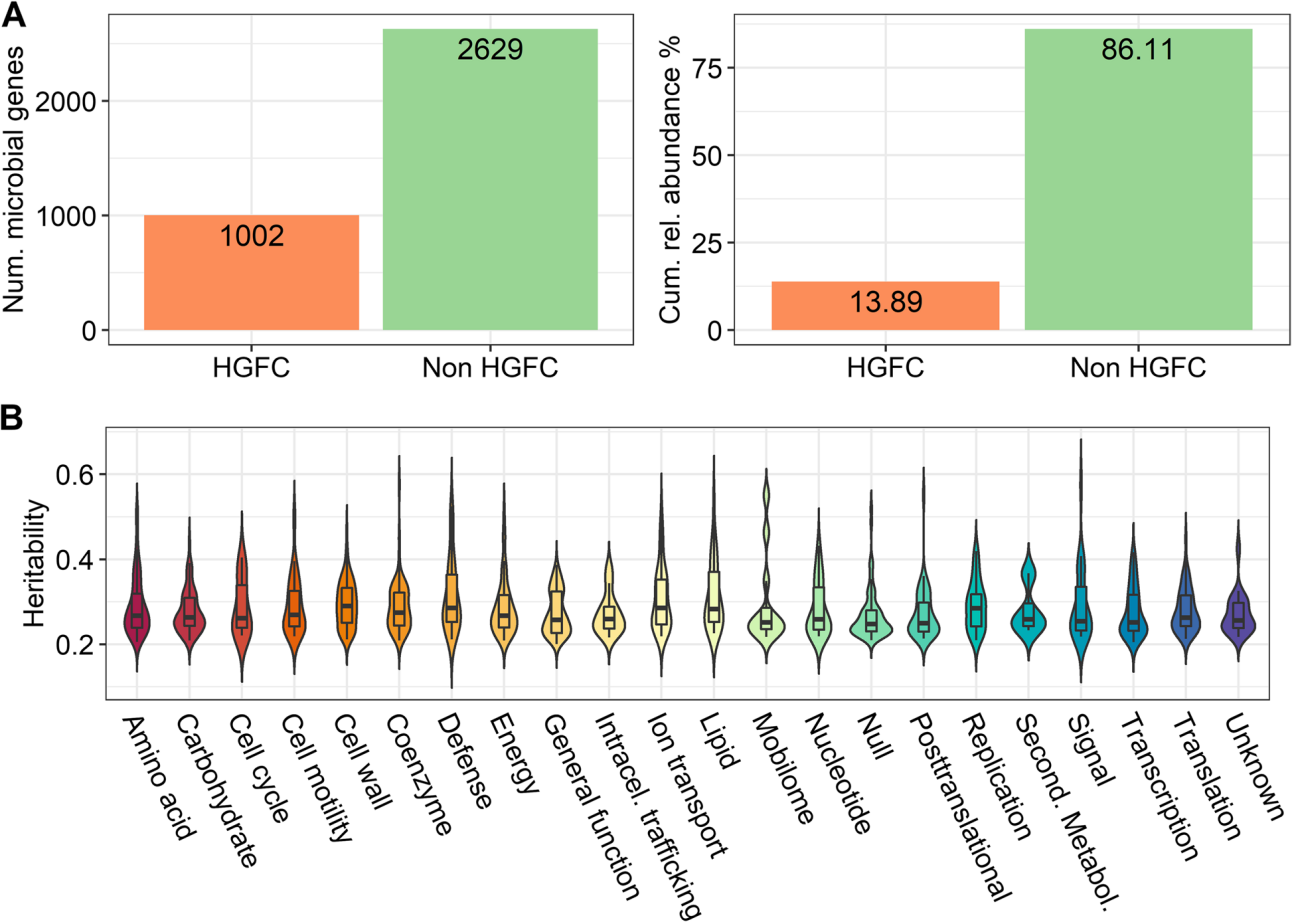
Host-genomically influenced functional core microbiome (HGFC) in the rumen of cattle identified as additive log-ratio transformed microbial gene abundances (*alr*-MGs) with ≥ 70% occupancy across animals and highly probable host genomic effects. **A** Number of *alr*-MGs and summed cumulative relative abundance of *alr*-MGs numerators comprehending the HGFC in our population. **B** Violin plots represent the distribution of heritability estimates for the 1002 HGFC *alr*-MGs, classified by COG functional modules of the numerators, represented by different colors. Full COG names are described in Table S11

To identify the part of the functional core microbiome (*n*=3631 *alr-*MGs with ≥70% occupancy) which was host-genomically influenced with strong evidence, we estimated the h^2^ of the *alr-*MGs and tested the significance of their host genomic effects by calculating the DIC_diff_ and BF. A joined condition of DIC_diff_ < −20 and BF > 14.5 was required to consider a host genomic influence on the abundance of each *alr-*MG. Our results indicate that 27.6% of the functional core microbiome (1002/3631 *alr-*MGs tested) is, at least, moderately heritable (*h*^2^ ranging from 0.20 to 0.58); the 1002 *alr*-MGs numerators together accounting for ~14% of the total relative abundance in the rumen (Fig. 1A and Table S1). This group of 1002 *alr*-MGs, advantageous for genomic selection, were referred to as the host-genomically influenced functional core microbiome (HGFC).

Only three *alr-*MGs encoding lipolytic activity on dietary lipids were part of the functional core microbiome (≥70% occupancy); *triacylglycerol lipase* (*lip*), *phospholipase A2/A1* (*pldA*) and *carboxylesterase* (*yvaK*) [20]. Their *h*^2^ estimates were in the range of 0.16–0.22 and only *pldA* (*h*^2^=0.22[0.00, 0.48]) was considered part of the HGFC (Table S2). Other *alr-*MGs encoding enzymes with biohydrogenation activity (*very long-chain enoyl-CoA reductase*, *alcohol-forming fatty acyl-CoA reductase*, *delta24-sterol reductase*, and *acyl-lipid n-6 desaturase*) were found in our database with a low occupancy rate (≤ 50% of animals) and were not considered part of the functional core microbiome. In contrast, *alr-*MGs involved in essential pathways, e.g. proteolysis and amino acid transport (e.g. *aroF* and *serA*), carbohydrate fermentation (*galT*) and transport (e.g. *msmX*) [62] or cell wall biosynthesis (e.g. *lpxB*, *lpxA*, *lptA*) were found to be heritable with an average *h*^2^ when classified within functional COGs around ~0.29 and HPD_95%_ of [0.2, 0.4] (Fig. 1B). Interestingly, the *alr-*MGs with the highest *h*^2^ in the rumen were related to signal transduction mechanisms (e.g. *glnB* in two-component system) and to vitamin B6 metabolism (*pdxT*), with *h*^2^ equal to 0.58 (Table S1).

### HGFC is associated to CLA and N3 indices in beef

Although *alr-*MGs directly involved in lipolytic and biohydrogenation activity appear to be not strongly influenced by the host genome, their metabolism has a strong interdependence (or present correlated responses) with other metabolic pathways, particularly those regulating H_2_ metabolism (e.g. carbohydrate degradation or microbial protein synthesis) [62], which were strongly influenced by the host genome. In our study, 372 of the 1002 *alr-*MGs comprising the HGFC were found to be involved in microbial metabolic pathways associated with N3 and/or CLA indices, as demonstrated by their strong host genomic correlations with either N3 (290 *alr-*MGs), CLA (66 *alr-*MGs) or both (16 *alr-*MGs) with *P*_0_ ≥ 0.95 (Table S3).

Our results suggest that the metabolism of amino acids and carbohydrates is potentially increased in the rumen of those hosts genetically determined to have a high N3 index. This is indicated by the strong and positive rg_N3_ (ranging from 0.53 to 0.87, *P*_0_≥0.95) estimated for the abundance of *alr-*MGs associated with the biosynthesis of phenylalanine, tyrosine and tryptophan (*aroF*, *aroD*, *enr*, *pheB*), metabolism of glycine, serine and threonine (*gcvPB*, *serA*, *thrH*, *grdD*), alanine (*gltD*, *TC.AGCS*, *alr*, *yafV*), thiamine (*iscS*, *tenA*, *thiD*, *thiI*) and cysteine and methionine (*E4.4.1.11*, *gshA*, *serA*, *speE*, *GTK*), ATP-binding cassette transporters of methionine (*metN*, *metI*, *metQ*) and the glutamate transport system (*gluD*). In addition, we also estimated strong and positive rg_N3_ for the abundance of *alr-*MG involved in putative aldouronate (*lplA*) and multiple sugar ABC transport systems (*msmX*, *ABC.MS.P1*, *ABC.MS.P*, *ABC.MS.S*), metabolism of fructose and mannose (*srlA*, *srlB*, *fruA*), starch and sucrose (*glgC*, *E2.4.1.4*, *celF*), galactose (*galT*, *gatB*, *sacA. malL*) and ascorbate and aldarate (*ulaA*, *lyxK*). CLA index was also related to amino acid and carbohydrate metabolism, as we estimated strong positive or negative rg_CLA_ (from |0.53| to |0.81|, *P*_0_≥0.95) with specific *alr-*MGs on these metabolic pathways, but lower in number. For example, *glxK* in the metabolism of glycine, serine and threonine, *ardD* and *hyuA* of arginine, *cap1J* of ascorbate and aldarate, *celC* and *glgA* of starch and sucrose, *glf* in galactose, or *pgi* and *tpiA* in glycolysis. Along with amino acid and carbohydrate metabolism, two microbial processes in HGFC were strongly associated with both N3 and CLA indices (Table S3). The first is translation, ribosomal biogenesis and transcription, with positive or negative r_g_ with N3 (*ybak*, *cca*, *cggR*, *hrpB*, *yedL*, *yafQ*, *fliA*, *parD1*, rg_N3_ =|0.46| to |0.81|, *P*_0_≥0.95) and CLA indices (*RP-S21*, *RP-S19*, RP-L35, *agar, tfoS,* rg_CLA_ =|0.62| to |0.78|, *P*_0_≥0.96). This result could reflect the modulation of microbial growth [63] in the presence or absence of specific long chain n-3 fatty acid and *cis-9*, *trans-11* C18:2 intermediates in the rumen [24]. The second microbial process is the metabolism of coenzymes, specifically folate, with e.g. *folC* and *pabBC* associated positively with CLA (rg_CLA_=0.66 and 0.64, *P*_0_=0.96), and *folk*, *folP*, *folB*, and *folE* associated negatively with N3 (rg_N3_ from −0.55 to −0.62, *P*_0_>0.96); or the metabolism of riboflavin (*ribE*, *ribH*, *yscE*), porphyrin (*cobN*, *pduO*), and pantothenate and CoA (*coaW*, *poK*), in which different *alr-*MGs were detrimental to both indices N3 (*ribE*, *ribH*, *coaW*, *cobH* and *pduO* showed rg_N3_=−0.54 and −0.69, *P*_0_>0.96) and CLA (*yscE*, *pok*, *cobN* showed rg_CLA_=−0.73, −0.65 and −0.70, *P*_0_>0.96).

**Fig. 2:**
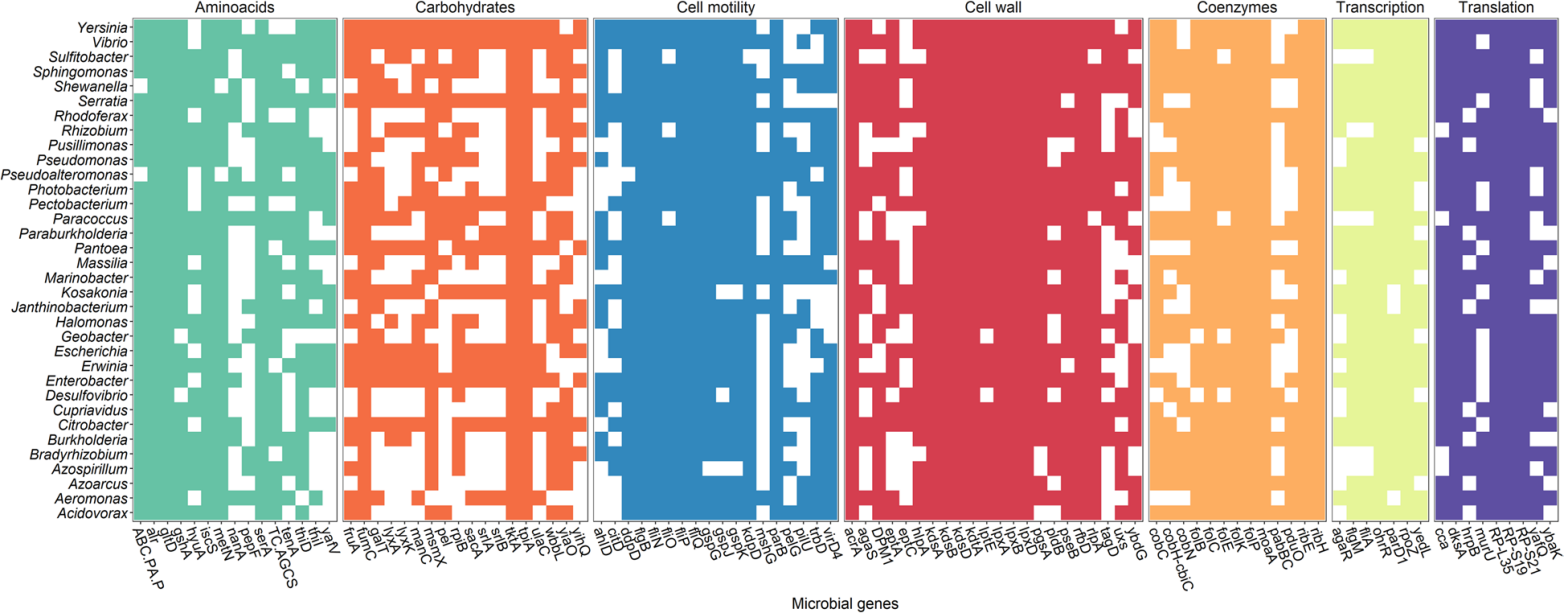
Microbial genomes of genera from the Proteobacteria phylum highly enriched in microbial genes genomically correlated with N3 and CLA indices. The number of microbial genes present in each microbial genus ranges from 70 (*Desulfovibrio*) to 96 (*Vibrio*) (see Table S4). Different colours represent different COG functional modules. Full COG names are described in Table S11

Another functional *alr-*MG group of the HGFC that was host-genomically correlated strongly with N3 index is linked to the biosynthesis of several metabolites of bacterial origin that function as cell membrane components: peptidoglycans (*pbpA*, *spoVD*, *dgkA*, *murT*, *vanY*), glycerophospholipids (*pgsA*, *tagD*, *pldB*), lipopolysaccharides (*kdsA*, *kdsB*, *kdsD*, *lpxD*, *lpxB*, *lpxA*, *lptA*, *kdtA*, *eptA*), lipoproteins (*lptE*, *rlpA*) and amino sugars and nucleotide sugars (*uxs*). Whereas *alr-*MGs involved in the biosynthesis of the first two cell wall components were positively correlated with the N3 index (rg_N3_ from 0.60 to 0.80, *P*_0_≥0.97), the abundances of *alr-*MGs in lipopolysaccharides, lipoproteins, and amino sugars and nucleotide sugars were negatively correlated (rg_N3_ from −0.43 to −0.70, *P*_0_≥0.95). Microbial mechanisms involved in cell motility were also strongly negatively associated to N3, with 6 *alr-*MGs for flagellar assembly and chemotaxis (*fliN*, *fliO*, *fliP*, *fliQ*, *flgB*, *flgM*), and one for pilus biogenesis (*mshG*) presenting rg_N3_ from −0.47 to −0.72 (*P*_0_≥0.96). Some of the abovementioned *alr-*MGs with strong rg_N3_ (e.g. *uxs*, *pdpA*, *ahId*, *fliN*, *fliO*, *fliP*, *fliQ*, *flgB*, *flgM*) were also host-genomically correlated with CLA index in the same direction as N3 index, but in lower magnitude and *P*_0_<0.95 (Table S3). To establish associations between microbial functions and the microbiota, we searched for the microbial genera identified in our animals carrying the largest number of the 372 identified *alr-*MGs according to KEGG database [64] (Fig. 2 and Table S4). Flagellar assembly *alr-*MGs involved either in the structural components or in the processing of environmental signals by chemotaxis (*fliN*, *fliO*, *fliP*, *fliQ*, *flgB*, *flgM*) were found extensively in the genome of several genera from the Proteobacteria phylum, many of them with lipolytic/biohydrogenation activity (*Pseudomonas*, *Vibrio*, *Aeromonas* and *Serratia*) [20, 21, 28]. Moreover, *gspG*, *gspK* and *gspJ*, associated with biofilm formation in *Vibrio* and *Pseudomonas* species (also carried by other Proteobacteria, see Table S4) followed the same pattern (rg_N3_ from −0.58 to −0.69, *P*_0_≥0.96).

### The influence of the host genome on the genera Anaerovibrio and Butyrivibrio and their genomic association with CLA and N3 indices

In our population, we investigated the influence of the host genome on the variation in abundance of the genera *Anaerovibrio* and *Butyrivibrio* (mean relative abundance of 0.15% and 2.54%), which are both well-known bacteria involved in lipolytic and biohydrogenation activity on dietary lipids in the rumen [62, 65, 66]. Our results suggest that the *clr*-transformed abundance of both genera were influenced by the host genome, but their *h*^2^ were low (*h*^2^=0.14 [0.00, 0.34] and *h*^2^=0.16 [0.00, 0.37], DIC_diff_ = −6.41 and −9.65, BF = 76 and 163). Despite its low *h*^2^, the *clr*-transformed abundance of *Anaerovibrio* genera was negatively host-genomically correlated with N3 index (rg_N3_=−0.75, *P*_0_=0.99), whilst its rg_CLA_ was lower (−0.30, *P*_0_=0.73). In contrast, the *clr*-transformed abundance of *Butyrivibrio* genus did not correlate strongly with the N3 or CLA indices, possibly because the abundance of different *Butyrivibrio* species play different roles in relation to biohydrogenation [67]. To increase resolution, we used information of two different uncultured *Butyrivibrio* strains (RUG14388 and RUG10859) identified at ≥70% occupancy in a subset our animals (*n*=282) by de novo metagenome-assembly of genomes [68]. Consistent with *Butyrivibrio* genus, their *h*^2^ values were low (*h*^2^=0.18 [0.00, 0.44] and 0.10 [0.00, 0.29], DIC_diff_ = −10.5 and −0.63, BF = 53 and 5.91); but interestingly, they showed different trends in their correlations with fatty acids indices, supporting our hypothesis. *Clr*-RUG14388 was negatively correlated only with CLA (rg_CLA_ = −0.82, *P*_0_=0.96) whilst *clr-*RUG10859 was strongly negatively correlated only with the N3 index (rg_N3_ = −0.82, *P*_0_=0.99).

### CLA and N3 indices in beef are simultaneously increased when genomic selection of the HGFC is applied

**Fig. 3:**
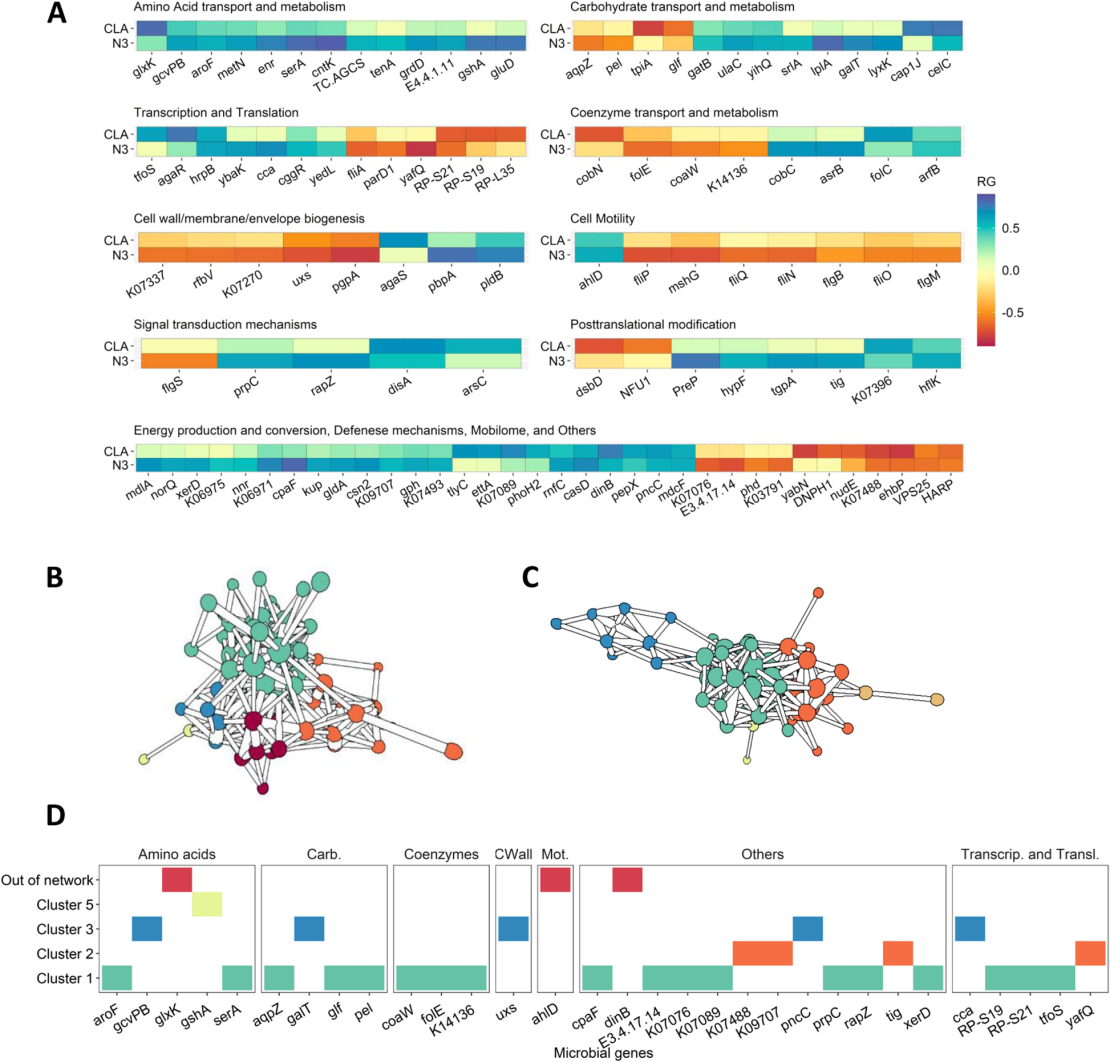
Study of 110 additive log-ratio transformed microbial gene abundances (*alr*-MGs) host-genomically influenced and correlated with N3 and CLA indices in the same direction. **A** Genomic correlations (RG) between *alr*-MGs and N3 and CLA indices in beef, classified by COG functional modules of the *alr*-MG numerators. Host genomic correlation estimates, and the names of the microbial genes are provided in Table S3. Co-abundance network analysis of **B** corrected phenotypic values or **C** estimated genomic breeding values for microbial gene abundances. Different colours indicate different clusters: 1 (green), 2 (orange) and 3 (blue). Edges correspond to the absolute Pearson correlation value between *alr*-MGs > l0.30l, and the thickness of the edges increases with the correlation size. The nodes represent *alr*-MGs and their size corresponds to the node degree (number of incident edges per node). **D** Thirty-one out of the 110 *alr*-MGs selected for breeding purposes classified along clusters and functions. Colours represent their position in the genomic co-abundance network analysis.

One of the objectives of this study was to design a microbiome-driven breeding strategy based on the variability of the *alr-*MGs comprising the part of the HGFC that is host-genomically correlated with N3 and CLA indices. Since we want to modify the microbial pathways that cause a simultaneous increase of both, we focused our research exclusively on the 110 of the 372 *alr-*MGs that have the same sign in their host genomic correlations mean with N3 (rg_N3_) and CLA (rg_CLA_) (Fig. 3A and Table S3).

As a first step, we analysed the phenotypic and host genomic correlation structure among the 110 *alr-*MGs in a co-abundance network based on phenotypic values (adjusted for fixed effects) and estimated genomic breeding values, respectively (Fig. 3B and C and Tables S5 and S6). Co-abundance networks are an accessible method that can shed light on ecological interactions [69] and potential keystones [70], although correlations may represent joint responses rather than direct interactions. In our study, the phenotypic and host genomic networks were constituted by 7 and 6 clusters, respectively, and both included three major co-abundance clusters (> 10 nodes or *alr-*MGs) with similar composition in both networks. Based on the host genomic network (Table S5 and Fig. 3B), *alr-*MGs in the same cluster are likely influenced by the same portion of the host genome. The three major clusters suggest a tight interaction or paired responses between *alr-*MGs in amino acid (*aroF*, *serA*, *grD*, *gcvPB*, *tenA*) and carbohydrate (*pel*, *lyxK*, *cap1J*, *glf*, *galT*, *srlA*, *tpiA*, *gatB*) metabolism and transport, folate (*folE*, *folC*) and pantothenate and CoA (*coaW*) biosynthesis, porphyrin metabolism (*cobC*), ribosomal biosynthesis (*RP-L35*, *RP-S19. RP-S21*), or flagellar assembly (*fliO*, *mshG*, *flgB*, *flgM*). In a second step, we aimed to select the smallest subset of *alr-*MGs that better explains the overall variability in the 110 *alr-*MGs suitable for breeding purposes. An imposed condition to include a *alr-*MG in our microbiome-driven breeding strategy was a minimum average relative abundance of the numerator MG across animals ≥0.01% (45 *alr-*MGs). Of these *alr-*MGs, we discarded those with redundant contribution (based on a redundancy analysis [71]) to explain the total variance contained in the host genomic breeding values of the 110 *alr-*MGs abundances and retained 31 *alr-*MGs that were significant (*P values* > 0.05, Fig. 3D and Table S7). The 31 selected *alr-*MGs covered most of the microbial processes highlighted in the discussion of this study and proportionately represented almost all clusters in the co-abundance network.

**Fig. 4:**
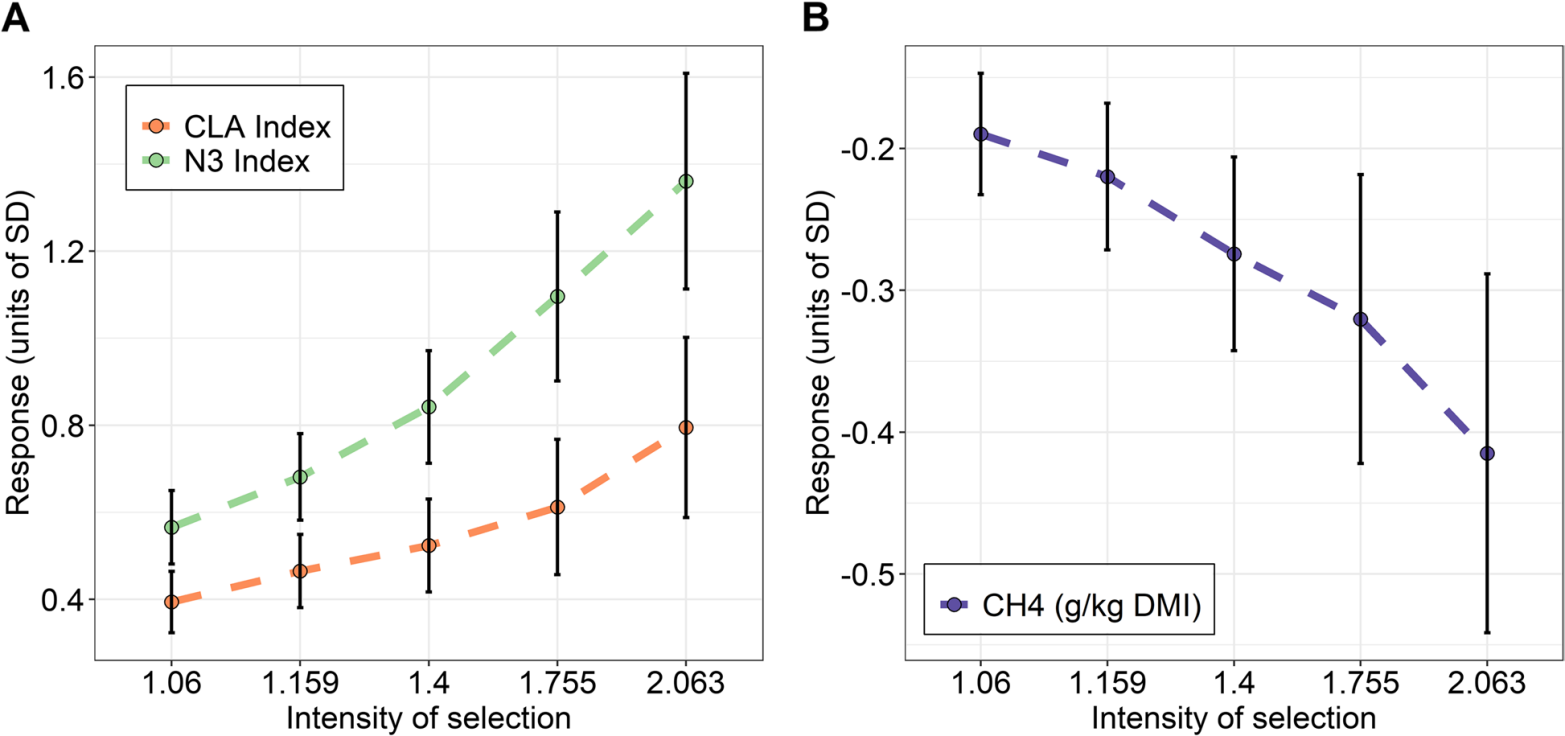
Responses to selection in **A** N3 and CLA indices and **B** methane emissions (CH_4,_ g/kg of dry matter intake) using the 31 additive log-ratio transformed microbial gene abundances (*alr*-MGs) as selection information (i.e., microbiome-driven breeding strategy). Responses to selection in CLA, N3 indices and CH_4_ emissions are estimated by selecting animals for their aggregate estimated breeding value for CLA and N3 (assuming equal economical weights) predicted using the 31 *alr*-MGs. Response is expressed in units of phenotypic standard deviations of the trait (SD)

Finally, we evaluated the accuracies and responses to selection achieved simultaneously in N3 and CLA indices based on the estimation of their host genomic breeding values by exclusively using the 31 *alr-*MGs as selection information. As a benchmark, we computed the CLA and N3 indices host genomic breeding values by using CLA or N3 indices observed data as a selection information. The mean estimation accuracies of genomic breeding values in the microbiome-driven breeding strategy were 0.65±0.027 for CLA and 0.65±0.028 for N3. The accuracies were lower than when using measured CLA or N3 indices as selection information (0.74±0.02 and 0.80±0.02, respectively). The microbiome-driven response to selection per generation in N3 and CLA, achieved by selecting animals of the analysed population with the highest aggregated breeding value for the goal traits (N3 and CLA indices) ranged depending on the selection intensity (from 1.06 to 2.06) between 0.57±0.08 and 1.36±0.24 phenotypic standard deviations (sd) for the N3 index (equivalent to 3.8 and 9.01% of the mean) and between 0.39±0.07 and 0.79±0.21 sd for the CLA index (equivalent to 4.9 and 9.82% of the mean) (Fig. 4A). These results demonstrate that a microbiome-driven breeding strategy based on the part of the HGFC associated to CLA and N3 is a suitable strategy to simultaneously improve fatty acid composition in beef while avoiding costly measurements of fatty acid content in beef.

### Mitigation of CH_4_ emissions is a consequence of genomic selection of the HGFC associated to CLA and N3 indices

**Fig. 5:**
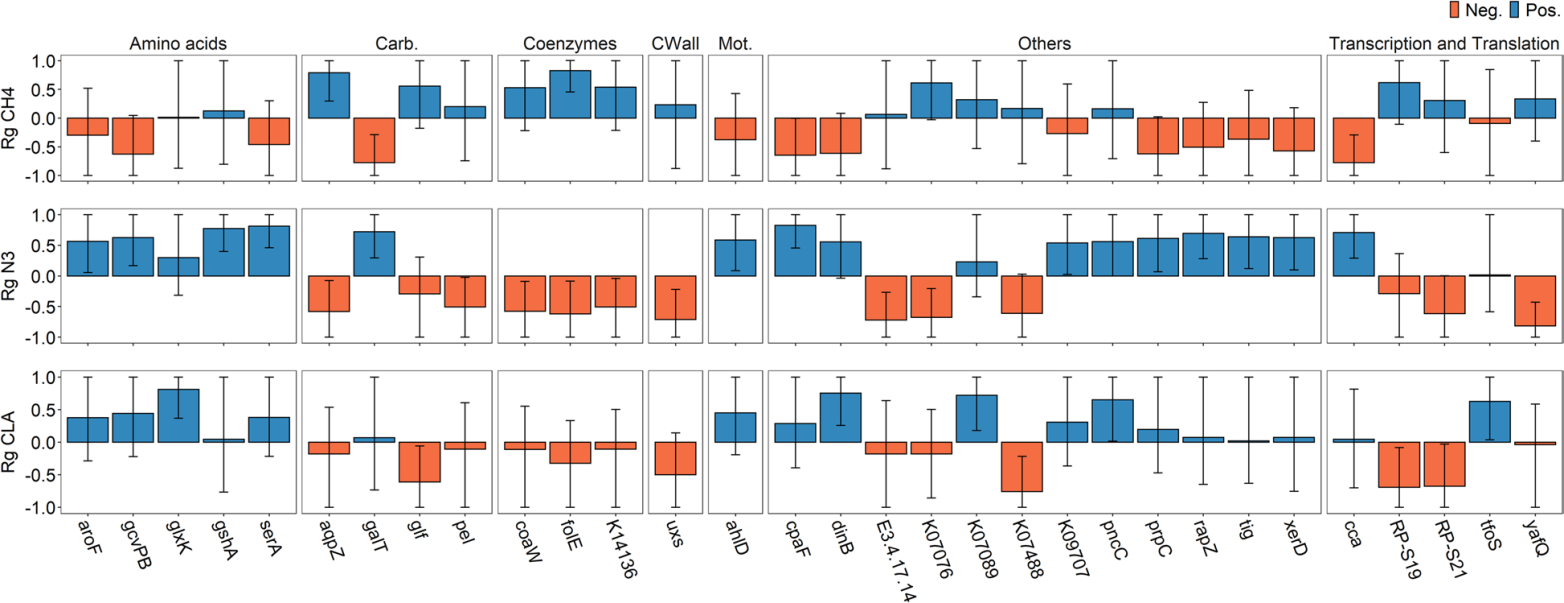
Host genomic correlations (Rg) between the 31 additive log-ratio transformed microbial gene abundances (*alr*-MGs) and CH_4_ (rg_CH4_), N3 (rg_N3_) and CLA (rg_CLA_) indices. The vast majority are favourable across traits, which indicate that an increase of CLA and N3 indices by genomic selection of the microbial gene abundances reduces CH_4_ emissions. Bars represent means and highest posterior density interval at 95% probability. For full names of *alr*-MGs, see Table S8

Targeting one microbial activity to the fatty acid composition in beef may have consequences for other traits such as CH_4_ emissions. Therefore, we estimated host genomic correlations between CH_4_ emissions and the 31 *alr-*MGs selected for the microbiome-breeding strategy to increase N3 and CLA (rg_CH4_, Table S8 and Fig. 5). Interestingly, almost all of these *alr-*MG abundances showed rg_CH4_ values with opposite (and therefore favourable) signs compared to rg_N3_ and rg_CLA_, 7 of them with *P*_0_>0.95. Finally, we estimated the consequence that selection on the aggregated breeding value for the goal traits (N3 and CLA indices), estimated exclusively based on information of the 31 *alr-*MG abundances, will have on CH_4_ emissions of the animals. This correlated response to selection on CH_4_ emissions ranged depending on selection intensity (1.06 to 2.06) from −0.19±0.04 to −0.41±0.12 sd of the CH_4_ trait or 4 to 9.4% of the mean per generation (Fig. 4B).

## Discussion

Variations in the hologenome to achieve improvement in production traits can be achieved by changes in either the host or microbiota genome [72]. In this study, we propose to apply host genomic selection targeting the microbial host-genomically influenced functional core microbiome in the rumen to approach a change in the hologenome towards an increased N3 and CLA indices in beef. Genomic selection requires the estimation of host genomic parameters, which we performed in this study using all genomic (386 samples), microbiome (359 samples) and fatty acids (245 animals) data available to obtain the most reliable estimates [73]. Moreover, all phenotypic and host genomic estimates presented in this study were obtained after adjusting observed traits for diet and other systematic effects including their interactions to focus genetic improvement on fatty acid variation unaffected by dietary intervention. The diets used in the experiments of the present study did not aim to change the fatty acid composition of beef, but to analyse the effects of diets on CH_4_ emissions. If rumen microbiome profiles within each diet showed the same increase in the trait, as shown by Roehe et al. [51] for CH_4_ emissions, it is reasonable to expect that the change due to diet intervention and microbiome-driven breeding would be additive. However, future specific dietary intervention experiments are needed to answer the question of whether the additivity of dietary intervention and microbiome-driven breeding also applies to the fatty acid profiles of beef.

We first investigated that ~28% of the ruminal functional core microbiome (1002/3633 *alr-*MGs tested) presented moderate to high *h*^2^ ranging from 0.20 to 0.58, which is sufficient to be efficiently targeted by host genomic selection. This result is similar to that obtained in our previous study [53] where 337/1141 or 29.5% of total *alr-*MGs identified in the rumen microbiome presented significant host genomic effects, with functional information now extended using an updated version of KEGG database and the bioinformatic KofamScan tool [74] (see the “Methods” section). The bacterial enzymes with lipolytic and biohydrogenation activity are well characterized in literature (e.g. lipases, phospholipases or isomerases) [15, 19–21]. In the same way, *Anaerovibrio lipolytica* and *Butyrivibrio*-like species are well-known bacteria involved in lipolytic and biohydrogenating activity on dietary lipids in the rumen [62, 65, 66]. An important result in our study is that inter-host variation in the *alr*-transformed abundance of MGs with potential lipolytic and biohydrogenation activity, and in the *clr*-transofrmed abundance of *Anaerovibrio* and *Butyrivibrio* genera, is likely to be more influenced by environmental factors rather than host genomic factors, with *h*^2^ ≤ 0.10 and host genomic effects that did not surpassed the stringent multitest significance threshold. Microbial metabolism of dietary lipids is a defence and/or adaptation mechanism of certain sensitive bacteria against UFA [15, 19, 22, 28, 35, 68, 75], but is not an essential activity of the microbial community, as bacteria are able to synthesise their own fatty acids, mainly for the construction of their cell membranes [76]. Instead, biohydrogenating and lipolytic activity appears to be a peripheral mechanism to the major growth-promoting activities, e.g. proteolysis and amino acid transport (e.g. *aroF* and *serA*), carbohydrate fermentation (*galT*) and carbohydrate transport (e.g. *msmX*) or cell wall biosynthesis (e.g. *lpxB*, *lpxA*, *lptA*), which was revealed to be under stronger host genomic influence (i.e. belonged to the HGFC).

In general, most metabolic processes in the rumen influence each other because of substrate interdependences, or because there are common metabolic pathways or common microbial species that carry out different processes, leading to joined responses. For example, *Butyrivibrio fibrisolvens* is a key player in fibre digestion, but many strains are also proteolytic and are involved in the biohydrogenation of fatty acids [77]. In our study, we found that N3 index in meat was strongly associated to 306 host-genomically influenced *alr-*MGs, whilst CLA is also influenced by HGFC, but to a lesser extent (82 *alr-*MGs). These results suggest that both N3 and CLA rely on HGFC microbial functions which indirectly regulate lipolysis and biohydrogenation rates in rumen. Additionally, these results highlight the possibility of applying genomic selection to the most informative *alr-*MG abundances in the HGFC to modify the fatty acid composition of meat by altering the flux of fatty acids available for intramuscular lipid accretion. The greater number of HGFC microbial functions linked to N3 than to CLA indicates that C18:3n-3 metabolism in rumen is susceptible to more diverse microbial actions than *cis-9*, *trans-11* C18:2 and its precursors. This is in line with the higher dependence of N3 than CLA on the host genome (*h*^2^_N3_=0.76±0.16 and h^2^_CLA_=0.57±0.17).

The positive host genomic correlation between N3 and CLA estimated in this study (rg_N3, CLA_=0.39) indicates the possibility of achieving a simultaneous increase in both indices by using genomic selection tools. Host genetics promoting a low rate of metabolism of dietary C18:3n-3 may also increase its concentration in the rumen, which is known from nutritional studies to impair survival of biohydrogenating bacteria [24], thereby improving the concentration of *trans-11* C18:1 and *cis-9*, *trans-11* C18:2 potentially available to be stored into body tissues [41]. Also, our study deciphers further host-genomically influenced microbial mechanisms that most likely contributed to the positive rg_N3,CLA_ as shown by 110 *alr-*MGs influencing N3 and CLA indices in the same direction. The metabolism of amino acids (*aroF*, *serA*, *glxK*) and carbohydrates (*gatB*, *ulaC*, *yihQ*, *galT*) are part of these HGFC mechanisms, positively associated to both indices (Fig. 3A). Biohydrogenation is particularly dependent on the metabolism of H_2_ [62], which is produced during the fermentation of sugars and then used in a number of processes such as microbial protein synthesis. The marginal H_2_ required for biohydrogenation of dietary lipids [78], along with the H_2_ resulting from enhanced carbohydrate metabolism in hosts with higher N3 and CLA, may be available for the synthesis of microbial proteins, most of which serve as amino acids for the host [79]. Dietary studies in which ruminants were fed with UFA also reported an increase in microbial protein synthesis, explained in part by defaunation [80–82], although interactions between protein and lipid metabolism in ruminants are far from being well understood. Another part of the HGFC mainly associated with N3,—but also to CLA index to some extent (see Fig. 3A)—was involved in the biosynthesis of several metabolites of bacterial origin that function as cell membrane components including peptidoglycans (*pbpA*, *spoVD*), glycerophospholipids (*tagD*, *pldB*), lipopolysaccharides (*kdsA*, *lpxD*, *lpxB*), lipoproteins (*lptE*, *rlpA*) and amino and nucleotide sugars (*uxs*). Since biohydrogenation of long chain n-3 fatty acids is a bacterial detoxification mechanism, these findings may be related to the disruption that their double bonds cause in the lipid bilayer structure of the bacterial membrane [22, 83]. On the other hand, an increased flux of lipopolysaccharides derived from the cell wall of Gram-negative bacteria in the rumen has been reported to downregulate the expression of stearyl-CoA desaturase in the host liver [84], thereby decreasing C16:1n-9/C16:0 and *cis-9*, *trans-11* C18:2/*trans-11* C18:1 desaturation indices in liver plasma [84]. The biosynthesis of lipopolysaccharides in the rumen could increase accumulation of C16:0 in the muscle, via the intestinal-liver axis or by inhibiting stearoyl-CoA desaturase expression in situ [85], potentially contributing to the impairment of both indices. However, in our study, *kdsA*, *lpxD* and *lpxB* showed strong and negative *r*_*g*_ with N3 but not with CLA index, which only partially supports this hypothesis. Another HGFC microbial process associated in the same direction with both N3 and CLA indices was cell motility. This was suggested by 7 *alr-*MGs involved either in the structural components or/and in the processing of environmental signals by chemotaxis (*fliN*, *fliO*, *fliP*, *fliQ*, *flgB*, *flgM*, *mshG*) and by 3 *alr-*MGs in biofilm formation (*gspG*, *gspK* and *gspJ*), all impacting negatively to healthy fatty acid indices in beef (see Fig. 3A). These *alr-*MGs were found extensively in the genome of several genera from the Proteobacteria phylum with lipolytic/biohydrogenation activity (*Pseudomonas*, *Vibrio*, *Aeromonas* and *Serratia*) [20, 21, 28]*.* An increased ability of these Proteobacteria genera to respond to changes in the rumen environment and migrate toward more desirable conditions, as well as to interact with other microbial communities [86], may favour their survival [87] and thus indirectly promote the metabolism of dietary lipids. Proteobacteria also produce acetate [88] (especially *Acetobacter*, *Kozaia*, *Asaia*, *Gluconobacter*, Table S3), which is the major precursor of de novo lipogenesis in ruminant adipose tissue [89]. The primary de novo lipogenesis product is C16:0 [90], which contributes to the increase in saturated fat content, although some of it can be elongated and unsaturated [90]. Another hypothesis linking chemotaxis to the impairment of N3 and CLA indices lies on the strong dependence of the final fatty acid flux available for absorption on the chemotaxis of some ciliates [22, 56, 62, 91–96]. A chemotaxis-dependent migration/sequestration mechanism selectively retains them in the rumen and lowers biomass of protozoa reaching the duodenum [94–96], which is a major loss, considering that their lipids are among the 30–43% of *cis-9*, *trans-11* C18:2 and the 40% of *trans-11* C18:1 [33] available for absorption, with also high contributions for long chain n-3 due to the engulfment of chloroplasts [97]. However, this hypothesis could not be investigated as none of the *alr-*MGs correlated to N3 and CLA involved in chemotaxis matched the genome of any protozoan genus in the KEGG database, and investigation of the links between protist genera and microbial functions in our database was hampered by the difficulty in identifying ciliate genera due to their low GC content [98, 99].

Rumen pH depends in part on host genetic factors, such as volatile fatty acid absorption by the epithelium [46], feeding behaviour—appetite, time and rhythm of chewing [100]—, the amount of saliva produced during chewing and the buffering capacity of saliva, which contributes to amortize ~30% of the acids produced daily in rumen. The enzymes of rumen bacteria involved in lipolysis and biohydrogenation have optimal activity at pH values between 7 and 9 [22]. Values of pH below 6 inhibit lipase activities (or lipolytic microorganisms, e.g. *Anaerovibrio lipolytica*) more than biohydrogenation [54], which at those pH values is less affected by the released of free long-chain UFA [62]. Given that the microbial mechanisms identified in our study vary with ruminal pH (e.g. the growth of species from the Proteobacteria phylum, microbial protein synthesis, carbohydrate metabolism, or lipopolysaccharide biosynthesis) [101–103], one might think that host genes associated with ruminal pH govern the changes described in this study. In our previous research [53], we identified the abundance of *alr-*MG *TSTA3* involved in the metabolism of host microbiome crosstalk mediator fucose [104] as an indicator of high pH values in the rumen (and consequently an indicator of high CH_4_ emissions). In this study, we estimated strong and negative (*P*_0_=0.99) rg_N3_ for *TSTA3* of −0.70 (whereas rg_CLA_ was not significant), which supports an increase in the metabolism of long-chain n-3 dietary lipids and decrease of N3 index at high ruminal pH values, and confirms the value of *TSTA3* as a ruminal pH indicator. However, pH may not be the only mechanism responsible. We have additionally proposed other microbiome mechanisms influenced by the host genome, such as acetate synthesized by Proteobacteria or lipopolysaccharide biosynthesis that are known to play key roles in bovine intramuscular fat biosynthesis [88, 89] and in the activity of stearoyl-CoA desaturase [84, 85].

The final objective in this study was to use the information contained in the 110 *alr-*MGs to predict the host genomic breeding values for N3 and CLA indices (i.e. microbiome-driven breeding strategy), while avoiding costly measurements of fatty acid content in beef. To facilitate the implementation of the MG information into genomic statistical methods, we studied their genomic covariance structure using co-abundance networks, which was greatly consistent with their covariance at phenotypic level, and used redundancy analysis to select a reduced group of 31 *alr-*MGs representative of the 110 *alr-*MGs. An additional imposed condition accomplished by the 31 *alr-*MGs was a minimum average relative abundance across animals ≥0.01%, as we preferred to avoid low counts as they are more prone to be absent in the rumen of some animals due to whole metagenomic sequence depth, and also because low counts could be subjected to more technical variation [105, 106]. Alternatively, a previous study in dairy cows used principal components to summarize microbiome information [44] to be integrated into breeding programs. This approach was promising, with microbiome-based principal components being heritable and host-genomically correlated with the trait of interest (CH_4_ emissions), although its interpretation is not straightforward, specifically when based on microbial functions. An advantage of our proposed selection strategy [53] is that it is based on host genomic correlations between *alr-*MG abundances and N3 and CLA indices which have biological meanings and can be easily interpreted and compared across studies. Our microbiome-driven breeding strategy presented prediction accuracies for N3 and CLA indices genomic breeding values which were reasonably close to those achieved when using measured CLA or N3 as selection information, and responses to selection in our population up to ~9% of the means of N3 and CLA indices, under 2.06 selection intensity. A direct consequence of our microbiome-driven breeding strategy is a permanent change in the HGFC composition, with enhanced or decreased abundances of the corresponding *alr-*MGs previously discussed. It is important to further study whether there are undesirable consequences of this microbiome change on other productive, environmental or health-associated traits of interest, which can be investigated by estimating the host genomic correlations between the *alr-*MGs used for breeding and the corresponding traits. In this study, we demonstrate that these secondary effects are not necessarily undesirable associated with the mitigation of CH_4_ emissions and thus with climate change. In fact, two of the 31 *alr-*MGs (*aroF* and *gshA*) are involved in the metabolism of cysteine and methionine or phenylalanine, tyrosine and tryptophan metabolic pathways. Certain *alr-*MGs on these metabolic pathways have been selected as highly informative for a microbiome-driven breeding strategy specifically designed to reduce CH_4_ emissions [53]. In this study, we estimated that a mitigation in CH_4_ emissions is expected (up to 9.4% of the mean of the trait under 2.06 selection intensity) when applying our microbiome-driven strategy to increase ~9% of N3 and CLA indices in beef. In nutritional studies, a substantial overlap has been observed between the inhibitory effects of long chain poly-UFA on biohydrogenation and methanogenesis [39, 55, 56], as they are toxic to both methanogens and protozoa [38]. On the other hand, biohydrogenation is probably not an alternative H_2_ sink for methanogenesis because the importance of biohydrogenation for the total H_2_ sink is very low, 1 to 2% of the H_2_ produced during fermentation is consumed by biohydrogenation [78]. H_2_ could instead be diverted to other beneficial routes for the host as the synthesis of microbial proteins, as shown in this study and also in our previous work [53].

## Conclusion

The high heritability and variation of the novel proposed N3 and CLA indices suggests that a healthier fatty acid profile in beef can be achieved through selective breeding. As an alternative to selection on this costly to measure fatty acid profiles in beef, we recommend an indirect selection strategy based on specific 31 rumen microbial functional genes which are host-genomically influenced and host genetically correlated with the fatty acid indices. This microbiome-driven breeding strategy is potentially more cost-effective and could achieved responses of up to 9% of the mean per generation in our population. Moreover, the 31 microbial functions had desirable host genomic correlations with CH_4_ emissions, and therefore, this breeding strategy would also have beneficial effect on CH_4_ mitigation. Furthermore, our study indicates that the host genomic link between the rumen microbiome and indicators of healthy fatty acids in the muscle is complex and does not necessarily rely on the well-known microbial genera or microbial functions with biohydrogenating and lipolytic actions. Instead, it comprised microbial metabolic pathways that indirectly affect microbial lipolytic and biohydrogenation activity, such as carbohydrate metabolism and microbial protein synthesis affecting H_2_ metabolism or cell membrane components biosynthesis that affects endogenous fatty acid synthesis in the muscle via stearoyl-CoA desaturase. Another example is microbial functions associated to flagellar biosynthesis, which are found in the genome of several genera from the Proteobacteria phylum. For implementation of the results into practise, the 31 microbial functions included in the microbiome-driven breeding strategy to increase healthy fatty acid indicators in beef could be combined with additional microbial functions specifically selected for reducing CH_4_ emissions and improving of feed conversion efficiency to develop an overall cost-effective novel breeding strategy based on the rumen microbiome that avoids the large costs associated with measuring those traits.

## Methods

### Animals

The data were obtained from 363 steers used in different experiments [107–111] conducted over 5 years (2011, 2012, 2013, 2014 and 2017). Animals were from different breeds (rotational crosses between Aberdeen Angus and Limousin breeds, Charolais-crosses and purebred Luing) and two basal diets consisting of 480:520 and 80:920 forage: concentrate ratios. Table S9 gives the distribution of the animals and data across experiments, breeds and diets. A detailed description of the diets can be found in [107–112]. A power analysis indicated that for the given number of animals per experiment, a genetic design of sires with on average 8 progeny per sire showed the highest power to identify genetic differences between sires.

### CH_4_ emission data

CH_4_ emissions were individually measured in 285 animals (Table S9) for 48h within six indirect open-circuit respiration chambers [110]. These animals represented the four breeds and both diets in a balanced design within (but not across) experiments: *n*~17 animals per diet and breed combination in the experiments performed in 2011, 2012 and 2013; and ~35 and 6 animals per breed in the experiments performed in 2014 and 2017, offered only forage-based diets. One week before entering the respiration chambers, the animals were housed individually in training pens, identical in size and shape to the pens inside the chambers, to allow them to adapt to being housed individually. At the time of entering the chamber, the average age of the animals was 528±38 days and the average live weight was 659±54 kg. In each experiment, the animals were allocated to the respiration chambers in a randomized design within breed and diet. Animals were fed once daily, and the weight of the feed offered and refused was recorded. CH_4_ emissions were expressed as g of CH_4_/kg of dry matter intake.

### Fatty acid data

The fatty acid composition of beef was available from 245 animals (Table S9). As before, these animals represented the four breeds and both diets in a balanced design within experiments: *n*~17 animals per diet and breed combination in the experiments performed in 2011 and 2013, *n*~10 in 2012 and ~35 animals per breed in 2014, offered only forage-based diets. At the time of slaughter, the animals were 567±22 days of age. After slaughter, the left carcass sides were cut at the 13th rib at 48 h post-mortem. After removing a 125-mm section from the caudal cut surface of *longissimus thoracis et lumborum*, the next 25 mm was taken, vacuum-packed and frozen for subsequent analysis. The fatty acid analysis was carried out at the University of Bristol by direct saponification [75, 113–115]. The samples were hydrolysed with 2 M KOH in water to methanol (1:1), and the fatty acids were extracted into petroleum spirit, methylated using diazomethane and analysed by gas liquid chromatography. The samples were injected in the split mode, 70:1, onto a CP Sil 88, 50 m ×0 .25 mm fatty acid methyl esters (FAME) column (Chrompack UK Ltd., London) with helium as the carrier gas. The output from the flame ionization detector was quantified using a computing integrator (Spectra Physics 4270, Santa Clara, CA, USA), and the linearity of the system was tested using saturated (FAME4) and monounsaturated (FAME5) methyl ester quantitative standards (Thames Restek UK Ltd., Windsor, Universal Biologicals (Cambridge) Ltd., Cambridge, UK), PUFA (n-3; Matreya, Universal Biologicals (Cambridge) Ltd., Cambridge, UK), short to medium chain and branched chain fatty acids (bacterial (bacterial acid methyl ester mix; Supelco, Sigma-Aldrich Company Ltd., Gillingham, UK) and a mix of C20 and C22 n-3 and n-6 fatty acids made in the laboratory from a mix of methyl esters (Sigma, Sigma-Aldrich Company Ltd., Gillingham, UK). Only major fatty acids are reported, representing over 90% of the total fatty acids present. C18:1*-trans* isomers are incompletely resolved by this procedure and are reported as one value. A full description of the meat fatty acid composition in our population is provided in Table S10.

### Collection and sequencing of genomic and metagenomic samples

For host DNA analysis, 6–10 ml of blood from the 363 steers was collected from the jugular or coccygeal vein in live animals or during slaughter in a commercial abattoir. Additional 7 blood and 23 semen samples from sires of the steers were available. The blood was stored in tubes containing 1.8 mg EDTA/ ml blood and immediately frozen to −20°C. Genomic DNA was isolated from blood samples using Qiagen QIAamp toolkit and from semen samples using Qiagen QIAamp DNA Mini Kit, according to the manufacturer’s instructions. The DNA concentration and integrity were estimated with Nanodrop ND-1000 (NanoDrop Technologies). Genotyping was performed by Neogen Genomics (Ayr, Scotland, UK) using GeneSeek Genomic Profiler (GGP) BovineSNP50k Chip (GeneSeek, Lincoln, NE). Missing SNP positions were imputed using Beagle 5.2 [116]. Genotypes were filtered for quality control purposes using PLINK version 1.09b [117]. SNPs were removed from further analysis if they met any of these criteria: unknown chromosomal location according to Illumina’s maps [118], call rates less than 95% for SNPs, deviation from Hardy-Weinberg proportions (*χ*^2^ test *P value*<10^−8^), or minor allele frequency less than 0.05. Animals showing genotypes with a call rate lower than 90% were also removed. After imputation and filtering, 386 animals and 38,807 SNPs remained for the analyses.

For microbial DNA analysis, post-mortem digesta samples (approximately 50 ml) from 363 steers were taken at slaughter (at 567±22 days of age) immediately after the rumen was opened to be emptied. Five milliliters of strained ruminal fluid was mixed with 10 ml of PBS containing glycerol (87%) and stored at −20 °C. DNA extraction from rumen samples was carried out following the protocol from Yu and Morrison [119] based on repeated bead beating with column filtration. DNA concentrations and integrity were evaluated by the same procedure (Nanodrop ND-1000) as for the blood samples. Four animals out of 363 did not yield rumen samples of sufficient quality for metagenomics analysis (therefore, we kept 359 samples). DNA Illumina TruSeq libraries were prepared from the entire genomic DNA of rumen samples and were whole metagenomic shotgun sequenced on Illumina HiSeq systems 4000 (samples from 283 animals from experimental years 2011, 2012, 2013 and 2014) [68, 120] or NovaSeq (samples from 76 animals from experimental year 2017) by Edinburgh Genomics (Edinburgh, Scotland, UK). Paired-end DNA reads (2 × 150 bp for Hiseq systems 400 and NovaSeq) were generated, resulting in between 7.8 and 47.8 GB per sample (between 26 and 159 million paired reads).

### Bioinformatics

To measure the abundance of known functional microbial genes, whole metagenome sequencing reads were quality trimmed using Fastp [121] and assembled using MEGAHIT [122]. Proteins were predicted using Prodigal [123], filtered to remove incomplete proteins and searched against the Kyoto Encyclopedia of Genes and Genomes (KEGG) database (https://www.genome.jp/kegg/ko.html) [124] (version 2020-10-04) using KofamScan [74]. Hits that passed KofamScan’s default thresholds were assigned to the KEGG orthologous groups (KO). Proteins that passed the threshold for multiple KOs were grouped separately, as were those that did not have a hit. The resulting KO grouping corresponded to a highly similar group of sequences. BWA-MEM [125] was used to map the reads against their assembly, and KO abundance was calculated using BamDeal [126] and BEDTools [127]. We identified a total of 7976 KO abundances, in this study referred to as microbial genes (MG). To discard those non-core microbiome functions, we used only MG present in at least 252 out of the 359 (70%) samples and mean relative abundance (RA) > 0.001%. This resulted in 3632 core MG cumulatively accounting for 99.55% of the total counts identified in the dataset.

For phylogenetic annotation of rumen samples, we followed the same pipeline as described in Martínez-Álvaro et al. [53]. Briefly, the sequence reads of 359 samples were aligned to a database including cultured genomes from the Hungate 1000 collection [128] and Refseq genomes [129] using Kraken software [130], and 1178 microbial genera abundances were identified. We kept only those microbes present in all the samples (to ensure sufficient data for the genomic analysis) and with a RA > 0.001% (1108 microbial genera), equivalent to 99.99% of the total number of counts. We used the 4941 rumen uncultured genomes (RUGs) identified by Stewart et al. [68] to identify and quantify the abundance of uncultured species in 282 animals of the present study. To ensure sufficient data for genomic analysis, we discarded those RUGs present in less than 70% of the animals (using a cutoff of 1X coverage) and kept 225 RUGs. The large number of RUGs discarded is related to the high specificity of the RUGs, which are often classified at the strain level and occur only in some of the animals. A detailed description of the metagenomics assembly and binning process and estimation of the depth of each RUG in each sample is described in Stewart et al. [68]. Our analysis was focused on the abundance of the two microbial genera *Butyrivibrio* and *Anaerovibrio*, present in the rumen samples of all animals, and of the two RUGs classified within the *Butyrivibrio* genera (RUG14388 and RUG10859) which were present in 204 of the 282 animals with RUG information.

### Log-ratio transformations of compositional data

We applied natural log-ratio transformations in fatty acids and microbiome data in order to deal with their compositional nature and mitigate the estimation of spurious correlations [131] between the traits.

### Fatty acid data

N3 [132] and CLA indices were calculated as follows:

$$N3=\ln \left(\frac{\mathrm{C}18:3\mathrm{n}-3+\mathrm{C}20:5\mathrm{n}-3+\mathrm{C}22:5\mathrm{n}-3+\mathrm{C}22:6\mathrm{n}-3}{\mathrm{C}12:0+\mathrm{C}14:0+\mathrm{C}16:0}\right)$$

$$CLA=\ln \left(\frac{cis-9, trans-11\ \mathrm{C}18:2+ trans-11\ \mathrm{C}18:1\ }{\mathrm{C}12:0+\mathrm{C}14:0+\mathrm{C}16:0}\right)$$

where individual fatty acid contents are expressed as grams per 100g of meat. Descriptive statistics of the indices in our beef population are provided in Table S10.

### Metagenomics data

Relative abundances equal to zero, which comprised 4.52% of the whole MG database, and 27.7% in the RUGs database, were imputed based on a Bayesian-multiplicative replacement by using *cmultrepl* function in zCompositions package [133]. This algorithm imputes zero values from a posterior estimate of the multinomial probability assuming a Dirichlet prior distribution with default parameters for GBM method [134] and performs a multiplicative readjustment of non-zero components to respect original proportions in the composition. Based on preliminary analyses, we found that additive natural log-ratio [71] transformation (*alr*) of the relative abundances of the MG (*alr*-MG), and a centred log-ratio [71] transformation (*clr*) of the relative abundances of microbial genera *Anaerovibrio* and *Butyrivibrio* and RUG14388 and RUG10859 were most appropriate log-ratio transformations for analysing the metagenomic data. Transformation *alr* permits comparison with other studies using different numbers of microbial parts in the total database (i.e. it has *subcompositional coherence*), and it has a simpler interpretation in practice than other natural log-ratio transformations (*clr* or isometric natural log-ratios), since they do not involved ratios of geometrical means [135]. Assuming *J* denotes the number of variables in the database (*J* =3632 MGs), the abundance of each *alr*-MG within a sample was expressed as [136]:

$$alr\left({x}_j\right)=\mathit{\ln}\left(\frac{x_j}{x_{ref}}\right)=\mathit{\ln}\left({x}_j\right)-\mathit{\ln}\left({x}_{ref}\right),\kern1.25em j=1,\dots, J-1,j\ne ref$$

where *x*_*j*_is the relative abundance of the *j*th MG (*j* from 1 to 3631) and *x*_*ref*_ is the relative abundance of a reference MG. The criteria to select the reference MG was a trade-off between a high Procrustes correlation between the exact log-ratio geometry and the approximate geometry engendered by the set of *alr-*MGs (i.e. maintains the isometry between samples), and a low variance of its log-transformed relative abundance, which further facilitates the *alr* interpretation reducing it to the numerator part [137] (Figure S2). The abundance of MG ribulose-phosphate 3-epimerase [EC:5.1.3.1] (*rpe*, KEGG code K01783) involved in pentose phosphate pathway (average relative abundance of 0.03% in our population) was selected as denominator with a high Procrustes correlation equal to 0.9974 and a small log-ratio variance equal to 0.0379 (coefficient of variation =5.08%, five points summary: min=−4.516, 1st quartile=−3.555, median=−3.466, mean =−3.502, 3rd quartile −3.405, max. −3.20). The small variation of *rpe* allowed us to simplify the interpretation of each *alr*-MGs as an interpretation of its numerator. Additionally, we selected *rpe* because it consistently presented these good properties in the microbiome composition of other ruminant MG data external to this study, including a longitudinal study (data not shown). From the biological point of view, *rpe*, which is involved in essential housekeeping functions, has been pointed as part of the core gene set along bacteria [138], and methanogenic archaea [139], and plays a key role in the sugar catabolism of several fungi [140]. In the case of the abundances of microbial genera *Anaerovibrio* and *Butyrivibrio* and RUG14388 and RUG10859, *alr* transformation was not used because, after a deep exploration, we did not find an appropriate reference microbial genera or RUG which strictly conserved the isometry between samples (Procrustes correlation was ≤ 0.96), and therefore, *clr* was used instead:

$$clr\left({x}_j\right)=\mathit{\ln}\left(\frac{x_j}{{\left(\prod_1^J{x}_j\right)}^{1/J}}\right)=\mathit{\ln}\left({x}_j\right)-\frac{1}{J}\sum_1^J\mathit{\ln}\left({x}_j\right),\kern0.5em j=1,\dots, J$$

Assuming *J* denotes the number of variables in the database (*J*=1108 in microbial genera and 225 in RUGs), and *x*_*j*_ the relative abundance of the *j*th MG.

### Study of diet, breed and experimental effects

To evaluate the magnitude of the combined effect of diet, breed and experiment (in order to account for all of these effects including their interactions which will be adjusted for in all genetic analysis) in CLA and N3 indices and the *alr* or *clr-*transformed whole functional and taxonomic microbiome databases, we used a one-way ANOVA analysis (N3, CLA indices) or one-way redundancy analysis (microbiome datasets) using R package vegan [141]. The combined effect of diet, breed and experiment explained 53% and 55% of the phenotypic variance of CLA and N3 indices (*P value* < 2.0×10^−16^). In microbiome databases, the combined effect accounted for 26.3% of the total variance in the *alr*-MG database, 28.6% in the microbial genera database comprised by *clr-Butyrivibrio* and *clr-Anaerovibrio* genera, and 32.9% in the RUGs database comprised by *clr-*RUG14388 and *clr-*RUG10859 (*P value* = 0.001 in all cases).

### Study of the host-genomically influenced functional core microbiome

A pipeline of the statistical analysis performed is displayed in Figure S3. Genomic heritabilities (*h*^2^) of 3631 *alr*-MGs, *clr-Anaerovibrio* and *clr-Butyrivibrio* genera, *clr-*RUG14388 and *clr-*RUG10859 abundances, CLA and N3 indices were estimated by fitting GBLUP univariate animal models including the combination of diet, breed and experiment as fixed effect (17 levels) and the host genomic effect, assumed to be normally distributed with mean equal to 0 and variance equal to the host genomic relationship matrix between the individuals [142] multiplied by the genomic variance. The genomic relationship matrix was built following the method 2 of Van Randen [142]. Residuals were assumed to be independently normally distributed and genomic and residual effects were assumed to be uncorrelated between them. Bayesian statistics were used [143], assuming bounded flat priors for all unknowns. Analyses were computed using the THRGIBBSF90 program [144]. Results were based on Markov chain Monte Carlo chains consisting of 1,000,000 iterations, with a burn-in period of 200,000, and to reduce autocorrelations, only 1 of every 100 samples was saved for inferences. In all analyses, convergence was tested using the POSTGIBBSF90 [144] programme by calculating the Z criterion of Geweke. Monte Carlo sampling errors were computed using time-series procedures and checked to be at least 10 times lower than the standard deviation of the marginal posterior distribution. As *h*^2^ estimates, we used the mean of its marginal posterior distribution and the highest posterior density interval at 95% probability (HPD_95%_). To test the significance of host genomic effects on each microbial trait, we analysed the data with and without genomic effects in the model and (i) compared the deviance information criterion [145] of the models and (ii) computed the Bayes Factor (using the approximation of Newton and Raftery [146]) as the ratio between the mean of the posterior likelihood distribution of the model with genomic effects divided by that of the model without genomic effects. We accounted for false discovery rate by setting a null hypothesis rejection threshold of Bayes Factor ≥14.5, following the procedure described in Wen et al. [147] assuming and alpha value of 0.0001, and estimating the upper-bond of parameter (proportion of the data generated from the null hypothesis) using the sample mean of the observed Bayes Factors [147]. Strong evidence of a host genomic effect on the microbial trait (i.e. belonging to the host-genomically influenced functional core microbiome (HGFC)) was defined when the joined condition of DIC of the full model being at least 20 points lower than the DIC in the reduced model, and a Bayes Factor higher ≥ 14.5. As generally accepted in animal breeding, we considered microbial abundances with *h*^2^ estimates <0.20 being lowly heritable, 0.20 < *h*^2^ < 0.40 being moderately heritable and *h*^2^ estimates >0.40 being highly heritable. Univariate analysis were also run from a frequentist approach using AIREMLF90 [144] software, and we obtained consistent heritability estimates (Pearson correlation between Bayesian and Frequentist heritability estimates was 0.88, and differences between both averaged −0.05±0.03).

### Host genomic correlations between HGFC and N3 (r_gN3_) and CLA (r_gCLA_) indices

We estimated the host genomic correlations between N3 or CLA index and the HGFC *alr*-MGs, *clr-Anaerovibrio* and *clr-Butyrivibrio* genera, *clr-*RUG14388 and *clr-*RUG10859 abundances. We fitted a GBLUP bivariate animal model per pairwise trait combination including the host genomic effect, normally distributed with a mean equal to 0 and variance equal to the Kronecker product of the genomic relationship matrix and the 2 × 2 host genomic (co)variance matrix of the 2 corresponding traits. Residuals were also assumed to be normally distributed with a mean equal to 0 and variance equal to the Kronecker product between an identity matrix of the same order as the number of individuals with data and the 2 × 2 residual (co)variance matrix of the 2 corresponding traits. Genomic and residual effects were assumed to be uncorrelated between them. Bayesian statistics were used, assuming bounded flat priors for all unknowns. Analyses were computed using the THRGIBBSF90 program. The results were based on Markov chain Monte Carlo chains consisting of 1,000,000 iterations, with a burn-in period of 200,000, and to reduce autocorrelations, only 1 of every 100 samples was saved for inferences. In all analyses, convergence was tested using the POSTGIBBSF90 [144] program by calculating the Z criterion of Geweke. Monte Carlo sampling errors were computed using time-series procedures and checked to be at least 10 times lower than the standard deviation of the marginal posterior distribution. As estimate for the host genomic correlations, we used the mean of its marginal posterior distribution and the HPD_95%_. To investigate their confidence level, we estimated the posterior probability of the host genomic correlation of being > or < 0 when the mean of the correlation was positive or negative (P_0_) and considered them significant when *P*_0_ ≥ 0.95.

### Microbial taxa associated to alr-MGs host-genomically correlated with fatty acid indices

To identify microbial taxa that have been found to contain the *alr-*MGs of interest (i.e. those belonging to the HFCM and host-genomically correlated with N3 and/or CLA indices) we used R (version 4.0.0) with the package KEGGREST (version 1.30) [64] to link the MGs with organism codes in the KEGG database. Codes beginning with RG were excluded as the corresponding taxa information was not available.

### Alr-MG co-abundance network analyses

We focus the following analysis exclusively on *alr-*MGs from the HGFC host-genomically correlated with CLA or/and N3 indices (*P*_0_ ≥ 0.95) and showing the same positive or negative correlation with both indices (*n*=110 *alr-*MGs). To study the phenotypic and host genomic correlation structure amongst the 110 *alr-*MGs, we computed a co-abundance network (Graphia software [148]) connecting or edging *alr-*MGs (nodes) when the Pearson correlation between their *alr* transformed abundances > l0.30l. We computed the co-abundance network at phenotypic and genomic level using as phenotype the pre-corrected data using the combination of diet, breed and experiment as fixed effect and as genotype the estimated genomic breeding values. The software applies Markov Clustering algorithm by a flow simulation model [149] to find discrete groups of nodes (clusters) based on their position within the overall topology of the graph. The granularity of the clusters, i.e. the minimum number of nodes that a cluster has to contain, was set to 2 nodes.

### Selection of alr-MG abundances for microbiome-breeding strategy and computation of their host genomic and residual co(variance) matrices

Of the 110 *alr-*MGs, we considered for breeding purposes only those with average relative abundances across animals ≥ 0.01% (*n*=45). We applied a redundancy analysis (R package vegan [141]) to test whether any of these 45 *alr-*MGs were redundant in explaining total variance contained in the estimated breeding values of the 110 *alr-*MGs. After discarding redundant *alr-*MGs (*P values* > 0.05), we kept 31 *alr-*MGs. To use *alr-*MG information to select hosts with increased N3 and CLA indices, the estimation between their host genomic and residual (co)variance matrices was required. Host genomic and residual (co)variances among the 31 selected *alr-*MGs were estimated using 465 bivariate analyses. Bivariate analyses fitted the same model as previously described for estimation of r_gN3_ and r_gCLA_ with the same assumptions. Results were based on Markov chain Monte Carlo chains consisting of 1,000,000 iterations, with a burn-in period of 200,000, and only 1 of every 100 samples was saved for inferences. Convergence was tested with POSTGIBBSF90 program by checking Z criterion of Geweke. Monte Carlo sampling errors were computed using time-series procedures and checked to be at least 10 times lower than the standard deviation of the posterior marginal distribution [143]. The 33 × 33 host genomic and residual variance-covariance matrices, including N3, CLA indices and the 31 *alr-*MGs were built with off-diagonals based on the means of the posterior distributions of the residual and genomic covariance estimates, and diagonals based on averaged means of posterior distributions of the residual and genomic variances estimates. Both matrices needed bending to be positive definite (tolerance for minimum eigenvalues=0.001). The difference between original and bent matrices was never higher than the posterior standard error of the corresponding parameters.

### Accuracy and response to the selection

We analysed two different scenarios to estimate the breeding value accuracies of N3 and CLA indices: (i) by using solely measured N3 or CLA index and (ii) by using the 31 *alr-*MGs. The two scenarios were computed with data from 245 animals with both fatty acid data and metagenomics information available to compare the two scenarios based on equal conditions. In both scenarios, estimated host genomic breeding values were calculated by GBLUP analysis assuming as fixed variance components the values on the previously estimated 33 × 33 host genomic and residual variance-covariance matrices of the traits after bending. Scenario (i) was performed using a univariate GBLUP analysis including only measured CLA or N3 index. The scenario (ii) was computed by fitting a multivariate GBLUP model including the 31 *alr-*MGs, CLA and N3 (setting CLA and N3 indices as missing values [150]). In these analyses, solutions were based on Markov chain Monte Carlo chains consisting of 100,000 iterations, with a burn-in period of 20,000, and to reduce autocorrelation only 1 of every 100 samples was saved for inferences. The accuracies of CLA and N3 indices estimated host genomic breeding values in each scenario were computed as:

11 $${Accuracy}_i=\sqrt{1-\frac{sd_i^2}{{g_{RM}}_{ii}\ast {\sigma}_g^2}}$$

where *sd*_*i*_ is the standard deviation of the posterior marginal distribution of the host genomic value for animal *i*, *g*_*RMii*_ is the genomic relationship matrix diagonal element for animal *i* and ${\sigma}_g^2$ is the genomic variance for N3 or CLA. The mean and standard deviation of the accuracies across animals was computed. To estimate the response to selection in N3 and CLA indices based on information from the 31 *alr-*MGs, we built the animal ranking based on the aggregated (sum) N3 and CLA estimated host genomic breeding values [150] assuming the estimated breeding values of each trait have equal economic weights as described in Schneeberger et al. [150]. Response to selection in N3 and CLA indices was estimated as the marginal posterior distribution of the difference between the mean estimated host genomic breeding values of all animals with data and the mean of selected animals based on the aggregated ranking when alternatively, 40%, 30%, 20%, 10% or 5% of our population were selected (equivalent to 1.06, 1.159, 1.4, 1.755 and 2.063 intensity of selection).

### Correlated responses in CH_4_ emissions after selection for the functional microbiome to improve N3 and CLA indices

First, we estimated the host genomic correlations between CH_4_ and the 31 *alr-*MGs following the same model and assumptions as previously described. Then, the estimated host genomic breeding values for CH_4_ emissions were obtained by microbiome-driven breeding after fitting a 33-multivariate GBLUP model setting CH_4_ observations as missing values and the 32 × 32 (31 *alr-*MGs and CH_4_ emissions) host genomic and residual variance-covariance matrices of the traits after bending as fixed variance components. Response to selection in CH_4_ emissions was estimated as the marginal posterior distributions of the difference between the mean of estimated host genomic breeding values of all animals with data and the mean of selected animals based on the previously defined aggregated ranking, when alternatively, 40%, 30%, 20%, 10% or 5% of our population were selected.

## Supplementary Information

**Additional file 1: Figure S1.** Distribution of N3 and CLA fatty acid indices in beef in our population. N3 index estimated as the natural logarithm of the ratio between C18:3n-3 + C20:5n-3 + C22:5n-3 + C22:6n-3 and C12:0 + C14:0 + C16:0. CLA index estimated as the natural logarithm of the ratio between *cis-9, trans-11* C18:2 + *trans-11* C18:1 and C12:0 + C14:0 + C16:0. Values are corrected by breed, diet and experiment combined effect. **Figure S2.** Selection of the microbial gene ribulose-phosphate 3-epimerase [EC:5.1.3.1] (*rpe,* KEGG code K01783) - highlighted in red - as a denominator for additive log-ratio transformation based on a balance between maximal Procrustes correlation with the complete pairwise log-ratio geometry and minimal log-ratio variance. **Figure S3.** Pipeline of the statistical analysis followed in the study. HGFC: host-genomically influenced functional core microbiome; MG: additive log-ratio transformed microbial gene abundances, h^2^: heritability estimate; CH_4_: methane: EBVs: host genomic breeding values; i: selection intensity.

**Additional file 2: Table S1.** Microbial gene abundances in rumen microbiome (analyzed after an additive log-ratio transformation) with significant host genomic effects referred to as host-specific functional core microbiome (HGFC). **Table S2.** Occupancy rates and heritabilities of micobial gene abundances (analyzed after an additive log-ratio transformation) involved in lipolysis and biohydrogenation processes in rumen found in our population. **Table S3.** Host genomic correlations between heritable additive log-ratio transformed microbial gene abundances and N3 and CLA Indices in beef with propbability of being positive of negative >95% (marked in bold). **Table S4.** The 963 different microbial genera in rumen carrying the 372 heritable additive log-ratio transformed microbial gene abundances both positively or negatively genomically correlated with N3 and CLA inidces in beef. **Table S5.** Composition of clusters from a co-abundance network analysis1 among phenotypic values (after correction for trial and diet) of the 110 additive log-ratio transformed microbial gene abundances genomically correlated with N3 and CLA indices with the same sign. **Table S6.** Composition of clusters from a co-abundance network analysis1 among estimated genomic breeding values of the 110 additive log-ratio transformed microbial gene abundances genomically correlated with N3 and CLA indices with the same sign. **Table S7.** Micorbial genes selected for breeding purpouses based on mean relative abundance (RA)>0.01%, significant genomic effects, host genomic correlation with N3 and CLA indices positively or negatively (P0 > 0.95) and significantly explaining part of the genomic variance inherent in the 110 additive-log transformed microbial genes. **Table S8.** Host genomic correlations between additive log-ratio transformed micorbial genes selected for breeding purpouses and methane emissions (g/kg dry matter intake). **Table S9.** Experimental design displaying the number of animals within each breed, diet and experiment. **Table S10.** Raw fatty acid composition (% of total fatty acids) and methane emissions (g/kg of dry matter intake) in beef cattle measured in 245 and 285 animals, respectively. **Table S11.** Correspondance between COG abreviations and full names.

## Data Availability

Metagenomic sequence reads for all rumen samples are available under the European Nucleotide Archive (ENA) under accession projects PRJEB31266, PRJEB21624 and PRJEB10338. The genotypes of the host animals are readily available from the authors.

## Abbreviations

N3 index: Natural log ratio between the sum of the long chain n-3 fatty acids (C18:3n-3 + C20:5n-3 + C22:5n-3 + C22:6n-3) and the sum of hypercholesterolemia saturated fatty acids (C12:0 + C14:0 + C16:0)
CLA index: Natural log ratio between the sum of *cis-9*, *trans-11* C18:2 + *trans*-11 C18:1 and the sum of hypercholesterolemia saturated fatty acids (C12:0 + C14:0 + C16:0)
CH_4_: Methane emissions expressed as g of CH_4_/kg of dry matter intake
MG: Microbial gene
*alr*: Additive log-ratio transformation
alr-MG: Additive log-ratio transformed microbial gene abundance
HGFC: Host-genomically influenced functional core microbiome
sd: Phenotypic standard deviation
BF: Bayes Factor
DIC_diff_: Deviance information criterion difference between models with and without genomic effects
RUGs: Rumen uncultured genomes

## References

[1] Simopoulos AP (2008). The importance of the omega-6/omega-3 fatty acid ratio in cardiovascular disease and other chronic diseases. Exp Biol Med (Maywood)..

[2] Brown WV, Karmally W, Kris-Etherton P, Rudel LR (2009). Discussion on dietary fat. J Clin Lipidol..

[3] Parodi PW (2009). Has the association between saturated fatty acids, serum cholesterol and coronary heart disease been over emphasized?. Int Dairy J..

[4] Parodi PW (2016). Dietary guidelines for saturated fatty acids are not supported by the evidence. Int Dairy J..

[5] Von Schacky C (2000). n-3 Fatty acids and the prevention of coronary atherosclerosis. Am J Clin Nutr..

[6] Calder PC (2006). n-3 Polyunsaturated fatty acids, inflammation, and inflammatory diseases. Am J Clin Nutr.

[7] Givens DI, Shingfield KJ (2004). Foods derived from animals: the impact of animal nutrition on their nutritive value and ability to sustain long-term health. Nutr Bull..

[8] Dalle Zotte A (2002). Perception of rabbit meat quality and major factors influencing the rabbit carcass and meat quality. Livest Prod Sci..

[9] Bessa RJB, Alves SP, Santos-Silva J (2015). Constraints and potentials for the nutritional modulation of the fatty acid composition of ruminant meat. Eur J Lipid Sci Technol..

[10] De Jager N (2013). Gene expression phenotypes for lipid metabolism and intramuscular fat in skeletal muscle of cattle. J Anim Sci..

[11] Ritzenthaler KL (2001). Estimation of conjugated linoleic acid intake by written dietary assessment methodologies underestimates actual intake evaluated by food duplicate methodology. J Nutr..

[12] Palmquist DL, Lock AL, Shingfield KJ, Bauman DE (2005). Biosynthesis of conjugated linoleic acid in ruminants and humans. Adv Food Nutr Res..

[13] Pariza MW (2004). Perspective on the safety and effectiveness of conjugated linoleic acid. Am J Clin Nutr..

[14] Smith SB (2006). Adiposity, fatty acid composition, and delta-9 desaturase activity during growth in beef cattle. Anim Sci J..

[15] Bauman DE, Baumgard LH, Corl BA, Griinari JM (1999). Biosynthesis of conjugated linoleic acid in ruminants. Proc Am Soc Anim Sci..

[16] Bauman DE (2000). Conjugated linoleic acid is synthesized endogenously in lactating dairy. J Nutr.

[17] Chilliard Y, Ferlay A, Bernard L, Rouel J, Doreau M (2007). Diet, rumen biohydrogenation and nutritional quality of cow and goat milk fat. Eur J Lipid Sci Technol.

[18] Toral PG, Hervás G, Leskinen H, Shingfield KJ, Frutos P (2018). In vitro ruminal biohydrogenation of eicosapentaenoic (EPA), docosapentaenoic (DPA), and docosahexaenoic acid ( DHA ) in cows and ewes: intermediate metabolites and pathways. J Dairy Sci..

[19] Buccioni A, Decandia M, Minieri S, Molle G, Cabiddu A (2012). Lipid metabolism in the rumen: New insights on lipolysis and biohydrogenation with an emphasis on the role of endogenous plant factors. Anim Feed Sci Technol..

[20] Privé F (2015). Isolation and characterization of novel lipases/esterases from a bovine rumen metagenome. Appl Microbiol Biotechnol..

[21] Arpigny JL, Jaeger K (1999). Bacterial lipolytic enzymes: classification and properties. Biochem J.

[22] Enjalbert F, Combes S, Zened A, Meynadier A (2017). Rumen microbiota and dietary fat: a mutual shaping. J Appl Microbiol..

[23] Henderson C (1971). A study of the lipase produced by Anaerovibrio lipolytica, a rumen bacterium. J Gen Microbiol..

[24] Maia MR (2010). Toxicity of unsaturated fatty acids to the biohydrogenating ruminal bacterium, Butyrivibrio fibrisolvens. BMC Microbiol.

[25] Kepler CR, Tucker WP, Tove SB (1970). Biohydrogenation of unsaturated fatty acids. IV. Substrate specificity and inhibition of linoleate delta-12-cis, delta-11-trans-isomerase from Butyrivibrio fibrisolvens. J Biol Chem..

[26] Kepler CR, Tove SB (1967). Biohydrogenation of unsaturated fatty acids. 3. Purification and properties of a linoleate delta-12-cis, delta-11-trans-isomerase from Butyrivibrio fibrisolvens. J Biol Chem..

[27] Kepler CR, Hirons KP, Mc Neil JJ, Tove SB (1966). Intermediates of linoleic and products of the biohydrogenation acid by Butyrivibrio fibrisolvens *. J Biol Chem..

[28] Heipieper HJ, Meinhardt F, Segura A (2003). The cis-trans isomerase of unsaturated fatty acids in Pseudomonas and Vibrio: biochemistry, molecular biology and physiological function of a unique stress adaptive mechanism. FEMS Microbiol Lett..

[29] Yokoyama MT, Davis CL (1971). Hydrogenation of unsaturated fatty acids by Treponema (Borrelia) strain B 2 5, a rumen spirochete. J Bacteriol..

[30] Wallace JR (2006). Clostridium proteoclasticum: A ruminal bacterium that forms stearic acid from linoleic acid. FEMS Microbiol Lett..

[31] Wright DE (1961). Bloat in cattle. XX. Lipase activity of rumen micro-organisms. New Zeal. J Agric Res..

[32] Coleman GS, Kemp P, Dawson RM (1971). The catabolism of phosphatidylethanolamine by the rumen protozoon Entodinium caudatum and its conversion into the N-(1-carboxyethyl) derivative. Biochem J..

[33] Yáñez-Ruiz DR, Scollan ND, Merry RJ, Newbold CJ (2006). Contribution of rumen protozoa to duodenal flow of nitrogen, conjugated linoleic acid and vaccenic acid in steers fed silages differing in their water-soluble carbohydrate content. Br J Nutr..

[34] Stergiadis S (2021). Unravelling the role of rumen microbial communities, genes, and activities on milk fatty acid profile using a combination of omics approaches. Front Microbiol..

[35] Maia M, Chaudhary L, Figueres L, Wallace J (2007). Metabolism of polyunsaturated fatty acids and their toxicity to the microflora of the rumen. Antonie Van Leeuwenhoek.

[36] Wallace JR (2008). Gut microbiology - broad genetic diversity, yet specific metabolic niches. Animal.

[37] Dewhurst RJ, Shingfield KJ, Lee MRF, Scollan ND (2006). Increasing the concentrations of beneficial polyunsaturated fatty acids in milk produced by dairy cows in high-forage systems. Anim Feed Sci Technol.

[38] Zhang CM (2008). Effect of octadeca carbon fatty acids on microbial fermentation , methanogenesis and microbial flora in vitro. Anim Feed Sci Technol.

[39] Goel G (2009). Effects of capric acid on rumen methanogenesis and biohydrogenation of linoleic and -linolenic acid. Animal.

[40] Hristov AN (2013). Special topics-Mitigation of methane and nitrous oxide emissions from animal operations: I. A review of enteric methane mitigation options. J Anim Sci..

[41] Toral PG, Monahan FJ, Hervás G, Frutos P, Moloney AP (2018). Review: modulating ruminal lipid metabolism to improve the fatty acid composition of meat and milk. Challenges and opportunities.

[42] Difford GF (2018). Host genetics and the rumen microbiome jointly associate with methane emissions in dairy cows. PLoS Genet..

[43] Zhang Q (2020). Bayesian modeling reveals host genetics associated with rumen microbiota jointly influence methane emission in dairy cows. ISME J..

[44] Saborío Montero A, et al. A dimensional reduction approach to modulate the core ruminal microbiome associated with methane emissions via selective breeding. J Dairy Sci. 2021. 10.3168/jds.2020-20005. 10.3168/jds.2020-20005 33896632

[45] Perlman D, et al. Concepts and consequences of a core gut microbiota to animal growth and development. Annu Rev Anim Biosci. 2021:1–25. 10.1146/annurev-animal-013020-020412. 10.1146/annurev-animal-013020-020412 34941382

[46] Li F (2019). Host genetics influence the rumen microbiota and heritable rumen microbial features associate with feed efficiency in cattle. Microbiome.

[47] Wallace JR (2019). A heritable subset of the core rumen microbiome dictates dairy cow productivity and emissions. Sci Adv..

[48] Saborío-Montero A (2020). Structural equation models to disentangle the biological relationship between microbiota and complex traits: Methane production in dairy cattle as a case of study. J Anim Breed Genet..

[49] Sasson G (2017). Heritable bovine rumen bacteria are phylogenetically related and correlated with the cow’s capacity to harvest energy from its feed. MBio.

[50] Weimer PJ, Stevenson DM, Mantovani HC, Man SLC (2010). Host specificity of the ruminal bacterial community in the dairy cow following near-total exchange of ruminal contents. J Dairy Sci..

[51] Roehe R (2016). Bovine host genetic variation influences rumen microbial methane production with best selection criterion for low methane emitting and efficiently feed converting hosts based on metagenomic gene abundance. PLoS Genet..

[52] Abbas W (2020). Influence of host genetics in shaping the rumen bacterial community in beef cattle. Sci Rep..

[53] Martínez-Álvaro M (2022). Bovine host genome acts on rumen microbiome function linked to methane emissions. Commun Biol..

[54] Van Nevel CJ, Demeyer DI (1996). Influence of pH on lipolysis and biohydrogenation of soybean oil by rumen contents in vitro. Reprod Nutr Dev..

[55] Martin C, Morgavi DP, Doreau M (2010). Methane mitigation in ruminants: from microbe to the farm scale. Animal.

[56] Jenkins TC, Wallace RJ, Moate PJ, Mosley EE (2008). Board-invited review: recent advances in biohydrogenation of unsaturated fatty acids within the rumen microbial ecosystem. J Anim Sci..

[57] Johnson PL (2021). Sheep divergently selected for methane yield showed differences in meat fatty acid composition. N Z J Anim Sci Prod.

[58] Gerber PJ (2013). Tackling climate change through livestock – a global assessment of emissions and mitigation opportunities.

[59] Eckard RJ, Grainger C, de Klein CAM (2010). Options for the abatement of methane and nitrous oxide from ruminant production: a review. Livest Sci..

[60] Myhre G, Shindell D, Bréon F-M, Collins W, Fuglestvedt J, Huang J, Koch D, Lamarque J-F, Lee D, Mendoza B, Nakajima T, Robock A, Stephens G, Takemura T, Zhang H. Anthropogenic and natural radiative forcing. In: Stocker TF, Qin D, Plattner G-K, Tignor M, Allen SK, Doschung J, Nauels A, Xia Y, Bex V, Midgley PM, Eds. Climate Change 2013: The Physical Science Basis. Contribution of Working Group I to the Fifth Assessment Report of the Intergovernmental Panel on Climate Change. Cambridge University Press; 2013. pp. 659–740. 10.1017/CBO9781107415324.018.

[61] Berry DP, Crowley JJ (2013). Cell biology symposium: genetics of feed efficiency in dairy and beef cattle. J Anim Sci..

[62] Lourenço M, Ramos-Morales E, Wallace RJ (2010). The role of microbes in rumen lipolysis and biohydrogenation and their manipulation. Animal.

[63] Tobin C (2011). Removal and replacement of ribosomal proteins.

[64] Tenenbaum D (2020). KEGGREST. Client-side REST acces to KEGG. Rpackage version 1.30.0.

[65] Hazlewood GP, Dawson RMC (1975). Isolation and properties of a phospholipid-hydrolising bacterium from ovine rumen fluid. J Gen Microbiol..

[66] Hazlewood G, Dawson RMC (1979). Characteristics of a lipolytic and fatty acid-requiring Butyrivibrio sp. isolated from the ovine rumen. J Gen Microbiol..

[67] Hussain SKA (2016). Characterization of CLA-producing Butyrivibrio spp. reveals strain-specific variations. 3 Biotech.

[68] Stewart RD (2019). Compendium of 4,941 rumen metagenome-assembled genomes for rumen microbiome biology and enzyme discovery. Nat Biotechnol..

[69] Coyte KZ, Rakoff-Nahoum S (2019). Understanding competition and cooperation within the mammalian gut microbiome. Curr Biol..

[70] Banerjee S, Schlaeppi K, van der Heijden MGA (2018). Keystone taxa as drivers of microbiome structure and functioning. Nat Rev Microbiol..

[71] Greenacre M (2019). Compositional data analysis in practise.

[72] Shapira M (2016). Gut microbiotas and host evolution: scaling up symbiosis. Trends Ecol Evol..

[73] Henderson CR (1975). Use of all relatives in intraherd prediction of breeding values and producing abilities. J Dairy Sci..

[74] Aramaki T (2020). KofamKOALA: KEGG Ortholog assignment based on profile HMM and adaptive score threshold. Bioinformatics.

[75] Prieto N (2010). Predicting beef cuts composition, fatty acids and meat quality characteristics by spiral computed tomography. Meat Sci..

[76] López-Lara IM, Geiger O (2010). Fomration of fatty acids. Handbook of Hydrocarbon and Lipid Microbiology.

[77] Stewart CS, Flint HJ, Bryant MP, Stewart CS, Hobson PN (1997). The rumen bacteria. in The Rumen Microbial Ecosystem.

[78] Nagaraja T, Newbold C, Van Nevel C, Demeyer D, Stewart PH (1997). Manipulation of ruminal fermentation. in The rumen microbial ecosystem.

[79] Pathak AK (2008). Various factors affecting microbial protein synthesis in the rumen. Vet World.

[80] Tesfa AT (1993). Effects of rape-seed oil supplementation on digestion, microbial protein synthesis and duodenal microbial amino acid composition in ruminants. Anim Feed Sci Technol..

[81] Broudiscou L, Pochet S, Poncet C (1994). Effect of linseed oil supplementation on feed degradation and microbial synthesis in the rumen of ciliate-free and refaunated sheep. Anim Feed Sci Technol..

[82] Ikwuegbu OA, Sutton JD (1982). The effect of varying the amount of linseed oil supplementation on rumen metabolism in sheep. Br J Nutr..

[83] Keweloh H, Heipieper HJ (1996). Trans unsaturated fatty acids in bacteria. Lipids.

[84] Xu T (2015). Lipopolysaccharide derived from the rumen down-regulates stearoyl-CoA desaturase 1 expression and alters fatty acid composition in the liver of dairy cows fed a high-concentrate diet. BMC Vet Res..

[85] Graugnard DE (2009). Adipogenic and energy metabolism gene networks in Longissimus lumborum during rapid post-weaning growth in Angus and Angus × Simmental cattle fed high-starch or low-starch diets. BMC Genomics.

[86] Won MY, Oyama LB, Courtney SJ, Creevey CJ, Huws SA (2020). Can rumen bacteria communicate to each other?. Microbiome.

[87] Nakamura S, Minamino T (2019). Flagella-driven motility of bacteria. Biomolecules.

[88] Kersters A, Lisdiyanti P, Komagata K, Swings J (2006). The family Acetobacteraceae: the genera Acetobacter, Acidomonas, Asaia, Gluconacetobacter, Gluconobacter and Kozaia. The Prokaryotes.

[89] Vernon RG (1980). Lipid metabolism in the adipose tissue of ruminant animals. Prog Lipid Res..

[90] Ladeira MM (2016). Nutrigenomics and beef quality: a review about lipogenesis. Int J Mol Sci..

[91] Shingfield KJ, Wallace RJ (2014). Synthesis of conjugated linoleic acid in ruminants and humans. RSC Catalysis Series 2014.

[92] Scollan ND, Price EM, Morgan SA, Huws SA, Shing KJ (2021). Conference on ‘ The future of animal products in the human diet: health and environmental concerns’ Symposium 1: meat, health and sustainability.

[93] Newbold CJ, De la Fuente G, Belanche A, Ramos-Morales E, McEwan NR (2015). The role of ciliate protozoa in the rumen. Front Microbiol..

[94] Abe M, Iriki T, Tobe N, Shibui H (1981). Sequestration of holotrich protozoa in the reticulo-rumen of cattle. Appl Environ Microbiol..

[95] Weller RA, Pilgrim AF (1974). Passage of protozoa and volatile fatty acids from the rumen of the sheep and from a continuous in vitro fermentation system. Br J Nutr..

[96] Diaz HL, Knapp JR, Karnati SKR, Dehority BA, Firkins JL (2014). Effects of wortmannin, sodium nitroprusside, insulin, genistein, and guanosine triphosphate on chemotaxis and cell growth of Entodinium caudatum, Epidinium caudatum, and mixed ruminal protozoa. J Dairy Sci..

[97] Huws SA (2009). Rumen protozoa are rich in polyunsaturated fatty acids due to the ingestion of chloroplasts. FEMS Microbiol Ecol..

[98] Chen YC, Liu T, Yu CH, Chiang TY, Hwang CC (2013). Effects of GC bias in next-generation-sequencing data on de novo genome assembly. PLoS One.

[99] Fliegerova K, Kaerger K, Kirk P, Voigt K, Puniya AK, Singh R, Kamra D (2015). Rumen Fungi. in Rumen Microbiology: From Evolution to Revolution.

[100] González LA, Manteca X, Calsamiglia S, Schwartzkopf-Genswein KS, Ferret A (2012). Ruminal acidosis in feedlot cattle: interplay between feed ingredients, rumen function and feeding behavior (a review). Anim Feed Sci Technol..

[101] Emmanuel DGV, Dunn SM, Ametaj BN (2008). Feeding high proportions of barley grain stimulates an inflammatory response in dairy cows. J Dairy Sci..

[102] Faniyi TO (2019). Role of diverse fermentative factors towards microbial community shift in ruminants. J Appl Microbiol..

[103] Castillo-Lopez E, Domínguez-Ordóñez MG (2019). Factors affecting the ruminal microbial composition and methods to determine microbial protein yield. Rev Mex Ciencias Pecu..

[104] Hooper LV, Gordon JI (2001). Glycans as legislators of host microbial interactions: spanning the spectrum from symbiosis to pathogenicity. Glycobiology.

[105] Fernandes DA, Macklaim JM, Linn TG, Reid G, Gloor GB (2013). ANOVA-Like Differential Expresion (ALDEx) Analysis for mixed population RNA-Seq. PLoS One.

[106] Quinn TP (2019). A field guide for the compositional analysis of any omics data. Gigascience.

[107] Duthie C-A (2016). Impact of adding nitrate or increasing the lipid content of two contrasting diets on blood methaemoglobin and performance of two breeds of finishing beef steers. Animal.

[108] Duthie CA (2018). The effect of dietary addition of nitrate or increase in lipid concentrations, alone or in combination, on performance and methane emissions of beef cattle. Animal.

[109] Duthie CA (2017). The impact of divergent breed types and diets on methane emissions, rumen characteristics and performance of finishing beef cattle. Animal.

[110] Rooke JA (2014). Hydrogen and methane emissions from beef cattle and their rumen microbial community vary with diet, time after feeding and genotype. Br J Nutr..

[111] Somarriba M (2019). The effects of a composite chronic stress treatment on fear responses and attention bias in beef cattle. ISAE 2019.

[112] Soley MAS (2020). The effects of stress on the microbial ruminal environment in beef cattle and its relationship to feed efficiency.

[113] Prieto N (2011). Online prediction of fatty acid profiles in crossbred Limousin and Aberdeen Angus beef cattle using near infrared reflectance spectroscopy. Animal.

[114] Teye GA (2006). Influence of dietary oils and protein level on pork quality. 1. Effects on muscle fatty acid composition, carcass, meat and eating quality. Meat Sci..

[115] Marley CL (2018). Stability, fatty acid composition and sensory properties of the M. Longissimus muscle from beef steers grazing either chicory/ryegrass or ryegrass. Animal.

[116] Browning BL, Zhou Y, Browning SR (2018). A one-penny imputed genome from next-generation reference panels. Am J Hum Genet..

[117] Purcell S (2007). PLINK: a tool set for whole-genome association and population-based linkage analyses. Am J Hum Genet..

[118] Matukumalli LK (2009). Development and characterization of a high density SNP genotyping assay for cattle. PLoS One.

[119] Yu Z, Morrison M (2004). Improved extraction of PCR-quality community DNA from digesta and fecal samples. Biotechniques.

[120] Stewart RD (2018). Assembly of 913 microbial genomes from metagenomic sequencing of the cow rumen. Nat Commun..

[121] Chen S, Zhou Y, Chen Y, Gu J (2018). Fastp: an ultra-fast all-in-one FASTQ preprocessor. Bioinformatics.

[122] Li D, Liu CM, Luo R, Sadakane K, Lam TW (2015). MEGAHIT: an ultra-fast single-node solution for large and complex metagenomics assembly via succinct de Bruijn graph. Bioinformatics.

[123] Hyatt D, Chen G-L, LoCascio PF, Land ML, Larimer FW, Hauser LJ (2010). Prodigal: prokaryotic gene recognition and translation initiation site identification. BMC Bioinformatics.

[124] Kanehisa M, Goto S (2000). KEGG: Kyoto Encyclopedia of Genes and Genomes. Nucleic Acids Res..

[125] Li H (2013). Aligning sequence reads, clone sequences and assembly contigs with BWA-MEM. BioArxiv.

[126] Barnett DW, Garrison EK, Quinlan AR, Strömberg MP, Marth GT. BamTools: a C++ API and toolkit for analyzing and managing BAM files. Bioinformatics. 2011;27(12):1691–2. 10.1093/bioinformatics/btr174. 10.1093/bioinformatics/btr174 PMC3106182 21493652

[127] Quinlan AR, Hall IM (2010). BEDTools: A flexible suite of utilities for comparing genomic features. Bioinformatics.

[128] Seshadri R (2018). Cultivation and sequencing of rumen microbiome members from the Hungate1000 Collection. Nat Biotechnol..

[129] Pruitt KD, Tatusova T, Maglott DR (2005). NCBI Reference Sequence (RefSeq): a cuarted non-redundant sequence database of genomes, transcripts and proteins. Nucleic Acids Res..

[130] Wood DE, Salzberg SL (2014). Kraken: ultrafast metagenomic sequence classification using exact alignments. Genome Biol..

[131] Greenacre M (2019). Compositional Data in practise.

[132] Perez R, Ca J, Dunner S (2010). Genes associated with long-chain omega-3 fatty acids in bovine skeletal muscle. J Appl Genet.

[133] Palarea-Albaladejo J, Martín-Fernández JA (2015). ZCompositions - R package for multivariate imputation of left-censored data under a compositional approach. Chemom Intell Lab Syst..

[134] Martín-Fernández JA, Hron K, Templ M, Filzmoser P, Palarea-Albaladejo J (2015). Bayesian-multiplicative treatment of count zeros in compositional data sets. Stat Modelling.

[135] Greenacre M (2018). Variable selection in compositional data analysis using pairwise logratios. Math Geosci..

[136] Aitchison J (1982). The statistical analysis of compositional data. J R Stat Soc Ser B(methodological).

[137] Greenacre M, Martínez-Álvaro M, Blasco A (2021). Compositional data analysis of microbiome and any-omics datasets: a revalidation of the additive logratio transformation. Front Microbiol..

[138] Gil R, Silva F, Pereto J, Moya A (2004). Determination of the core of a minimal bacterial gene set supplemental material for this article may be found at http://mmbr. asm. org. Microbiol Mol Biol Rev..

[139] Grazziotin AL, Vidal NM, Venancio TM (2015). Uncovering major genomic features of essential genes in Bacteria and a methanogenic Archaea. FEBS J..

[140] Khosravi C, Benocci T, Battaglia E, Benoit I, de Vries RP (2015). Sugar catabolism in Aspergillus and other fungi related to the utilization of plant biomass. Adv Appl Microbiol.

[141] Oksanen J (2020). vegan: Community Ecology Package. R package version 2.5-7.

[142] VanRaden PM (2008). Efficient methods to compute genomic predictions. J Dairy Sci..

[143] Blasco A (2017). Bayesian data analysis for animal scientists: the basics.

[144] Misztal I (2018). Manual for BLUPF90 family of programs.

[145] Spiegelhalter DJ, Best NG, Carlin BP, Van Der Linde A (2002). Bayesian measures of model complexity and fit. J R Stat Soc Ser B Stat Methodol..

[146] Newton MA, E., R. A. (1994). Approximate Bayesian inference with the weighted likelihood bootstrap. J R Stat Soc Ser B (Statistical Methodol).

[147] Wen X (2017). Robust Bayesian FDR control using bayes factors, with applications to multi-tissue eQTL discovery. Stat Biosci..

[148] Freeman TC, et al. Graphia: a platform for the graph-based visualisation and analysis of high dimensional data. Plos Comput Biol. 2022;18(7):e1010310. 10.1371/journal.pcbi.1010310 PMC9352203 35877685

[149] Freeman TC (2007). Construction, visualisation, and clustering of transcription networks from microarray expression data. PLoS Comput Biol..

[150] Schneeberger M, Barwick SA, Crow GH, Hammond K (1992). Economic indices using breeding values predicted by BLUP. J Anim Breed Genet..

