# Correction: Microbiome-driven breeding strategy potentially improves beef fatty acid profile benefiting human health and reduces methane emissions

**DOI:** 10.1186/s40168-022-01392-y

**Published:** 2022-10-25

**Authors:** Marina Martínez-Álvaro, Jennifer Mattock, Marc Auffret, Ziqing Weng, Carol-Anne Duthie, Richard J. Dewhurst, Matthew A. Cleveland, Mick Watson, Rainer Roehe

**Affiliations:** 1 Scotland’s Rural College, Edinburgh, UK; 2 The Roslin Institute and the Royal (Dick) School of Veterinary Studies, University of Edinburgh, Edinburgh, UK; 3 Agrifirm, Drongen, Belgium; 4 Genus plc, DeForest, WI USA

**Correction: Microbiome 10, 166 (2022)**

**https://doi.org/10.1186/s40168-022-01352-6**

Following the publication of the original article [1], the author reported that Prof. Rainer Roehe was not captured as co-corresponding author.

This has been corrected in this article and the original article has been updated.
