# Supplementary material for: Microbiome-driven breeding strategy potentially improves beef fatty acid profile benefiting human health and reduces methane emissions
Source: Microbiome. 2022 Oct 5;10:166. doi: 10.1186/s40168-022-01352-6 (PMC9533493; doi:10.1186/s40168-022-01352-6)
Supplement: Supplementary file 2 — Additional file 1: Figure S1. Distribution of N3 and CLA fatty acid indices in beef in our population. N3 index estimated as the natural logarithm of the ratio between C18:3n-3 + C20:5n-3 + C22:5n-3 + C22:6n-3 and C12:0 + C14:0 + C16:0. CLA index estimated as the natural logarithm of the ratio between cis-9, trans-11 C18:2 + trans-11 C18:1 and C12:0 + C14:0 + C16:0. Values are corrected by breed, diet and experiment combined effect. Figure S2. Selection of the microbial gene ribulose-phosphate 3-epimerase [EC:5.1.3.1] (rpe, KEGG code K01783) - highlighted in red - as a denominator for additive log-ratio transformation based on a balance between maximal Procrustes correlation with the complete pairwise log-ratio geometry and minimal log-ratio variance. Figure S3. Pipeline of the statistical analysis followed in the study. HGFC: host-genomically influenced functional core microbiome; MG: additive log-ratio transformed microbial gene abundances, h2: heritability estimate; CH4: methane: EBVs: host genomic breeding values; i: selection intensity. [file 40168_2022_1352_MOESM1_ESM.docx]

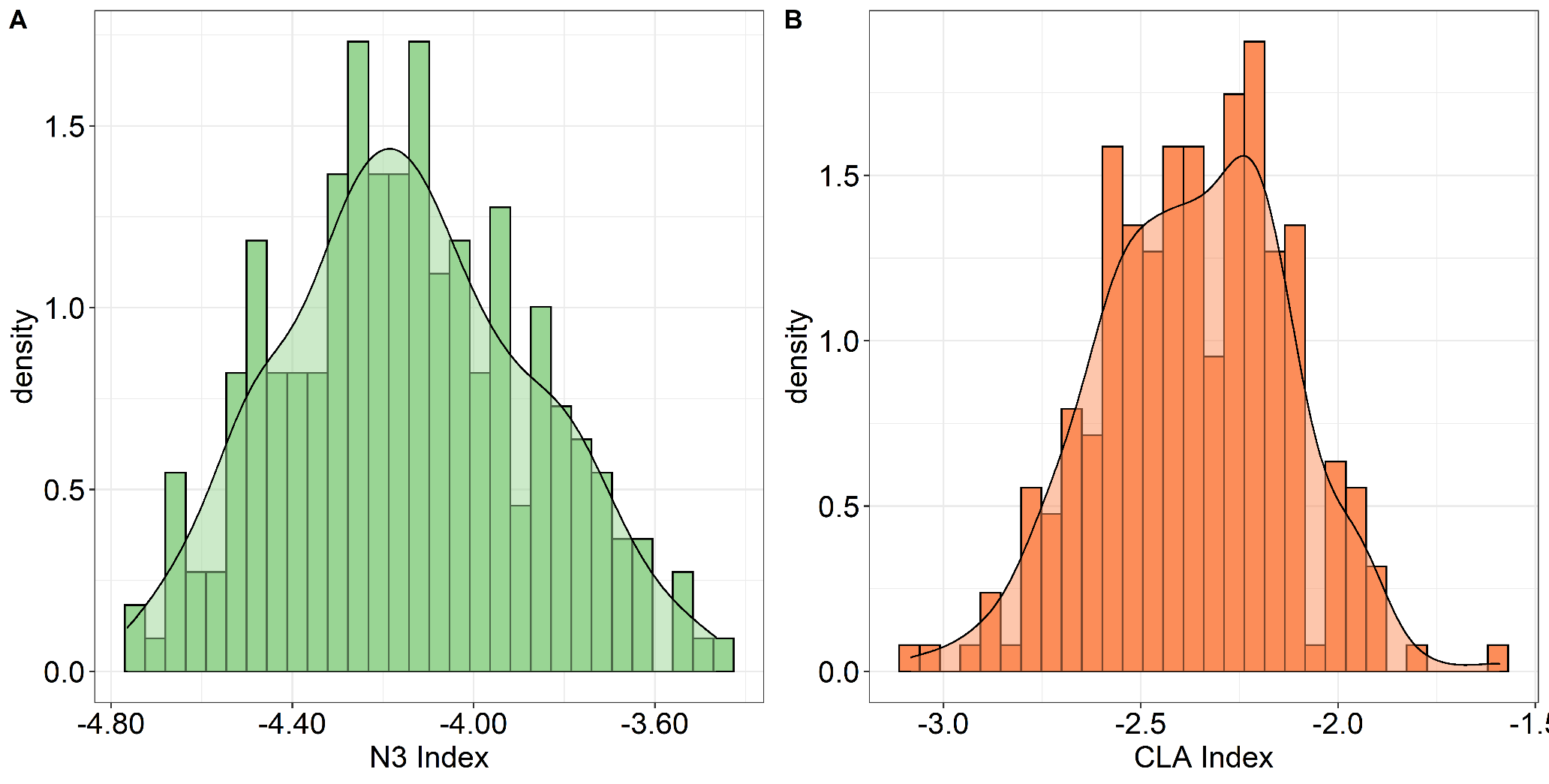


**Figure S1.** Distribution of N3 and CLA fatty acid indices in beef in our population. N3 index estimated as the natural logarithm of the ratio between C18:3n-3 + C20:5n-3 + C22:5n-3 + C22:6n-3 and C12:0 + C14:0 + C16:0. CLA index estimated as the natural logarithm of the ratio between *cis-9, trans-11* C18:2 + *trans-11* C18:1 and C12:0 + C14:0 + C16:0. Values are corrected by breed, diet and experiment combined effect.


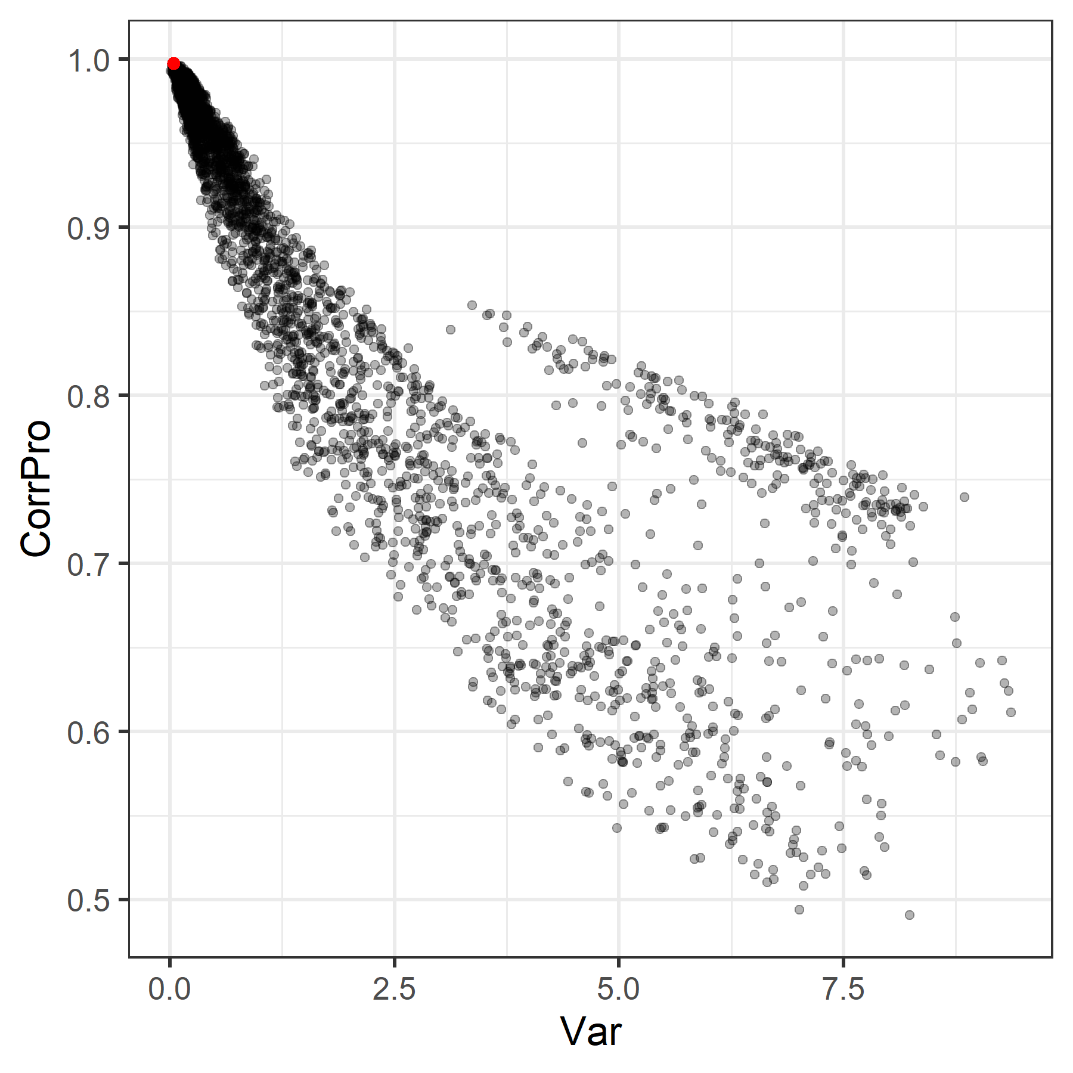


**Figure S2.** Selection of the microbial gene ribulose-phosphate 3-epimerase [EC:5.1.3.1] (*rpe,* KEGG code K01783) - highlighted in red - as a denominator for additive log-ratio transformation based on a balance between maximal Procrustes correlation with the complete pairwise log-ratio geometry and minimal log-ratio variance.


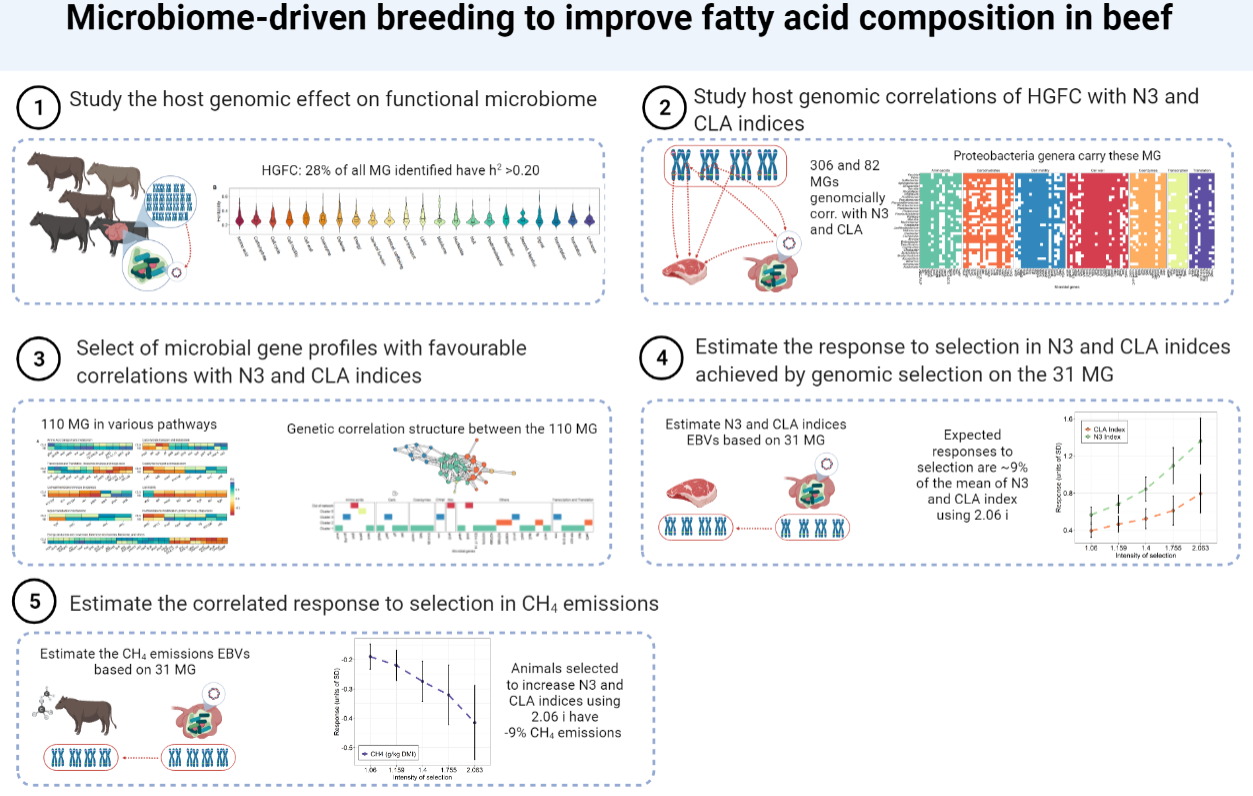


**Figure S3.** Pipeline of the statistical analysis followed in the study. HGFC: host-genomically influenced functional core microbiome; MG: additive log-ratio transformed microbial gene abundances, h^2^: heritability estimate; CH_4_: methane: EBVs: host-genomic breeding values; i: selection intensity.
